# ProMetIS, deep phenotyping of mouse models by combined proteomics and metabolomics analysis

**DOI:** 10.1038/s41597-021-01095-3

**Published:** 2021-12-03

**Authors:** Alyssa Imbert, Magali Rompais, Mohammed Selloum, Florence Castelli, Emmanuelle Mouton-Barbosa, Marion Brandolini-Bunlon, Emeline Chu-Van, Charlotte Joly, Aurélie Hirschler, Pierrick Roger, Thomas Burger, Sophie Leblanc, Tania Sorg, Sadia Ouzia, Yves Vandenbrouck, Claudine Médigue, Christophe Junot, Myriam Ferro, Estelle Pujos-Guillot, Anne Gonzalez de Peredo, François Fenaille, Christine Carapito, Yann Herault, Etienne A. Thévenot

**Affiliations:** 1 CEA, LIST, Laboratoire Sciences des Données et de la Décision, IFB, MetaboHUB, Gif-sur-Yvette, France; 2 IFB-core, UMS3601, Genoscope, Evry, France; 3 Laboratoire de Spectrométrie de Masse BioOrganique, Université de Strasbourg, CNRS, IPHC UMR 7178, ProFI, Strasbourg, France; 4 Université de Strasbourg, CNRS, INSERM, Institut Clinique de la Souris, Phenomin-ICS, Illkirch, France; 5 Université Paris Saclay, CEA, INRAE, Département Médicaments et Technologies pour la Santé (MTS), MetaboHUB, Gif-sur-Yvette, France; 6 Institut de Pharmacologie et Biologie Structurale (IPBS), Université de Toulouse, CNRS, UPS, ProFI, Toulouse, France; 7 Université Clermont Auvergne, INRAE, UNH, Plateforme d’Exploration du Métabolisme, MetaboHUB, Clermont-Ferrand, France; 8 CEA, LIST, Laboratoire Intelligence Artificielle et Apprentissage Automatique, MetaboHUB, Gif-sur-Yvette, France; 9 Université Grenoble Alpes, INSERM, CEA, UMR BioSanté U1292, FR2048, ProFI, Grenoble, France; 10 Laboratoire d’Analyses Bioinformatique en Génomique et Métabolisme (LABGeM), CNRS & CEA/DRF/IFJ, UMR8030 Evry, France; 11 Université de Strasbourg, CNRS, INSERM, Institut de Génétique Biologie Moléculaire et Cellulaire, IGBMC, Illkirch, France

**Keywords:** Proteomics, Metabolomics, Data integration, Mechanisms of disease

## Abstract

Genes are pleiotropic and getting a better knowledge of their function requires a comprehensive characterization of their mutants. Here, we generated multi-level data combining phenomic, proteomic and metabolomic acquisitions from plasma and liver tissues of two C57BL/6 N mouse models lacking the *Lat* (linker for activation of T cells) and the *Mx2* (MX dynamin-like GTPase 2) genes, respectively. Our dataset consists of 9 assays (1 preclinical, 2 proteomics and 6 metabolomics) generated with a fully non-targeted and standardized approach. The data and processing code are publicly available in the *ProMetIS* R package to ensure accessibility, interoperability, and reusability. The dataset thus provides unique molecular information about the physiological role of the *Lat* and *Mx2* genes. Furthermore, the protocols described herein can be easily extended to a larger number of individuals and tissues. Finally, this resource will be of great interest to develop new bioinformatic and biostatistic methods for multi-omics data integration.

## Abstract (summary)

**Table:** 

Measurement(s)	preclinical phenotype • protein expression profiling • metabolite profiling
Technology Type(s)	phenotyping tests • mass spectrometry assay
Factor Type(s)	genotype • sex
Sample Characteristic - Organism	Mus musculus

Machine-accessible metadata file describing the reported data: 10.6084/m9.figshare.15015273

## Background & Summary

The large scale analysis of gene function ongoing in the International Mouse Phenotyping consortium (www.mousephenotype.org) has consolidated the pleiotropic hypothesis of gene function in mammals^1–3^. Thus, more comprehensive approaches are needed to investigate gene’s function. The global study of proteins^4^ and metabolites^5,6^ are two major approaches for the understanding of biological processes and metabolism. Moreover, metabolites interact closely with proteins in the cell, either as substrates, cofactors and enzyme products, or as allosteric regulators of enzymes, transmembrane receptors or transcription factors^7^. Joint proteomic and metabolomic characterization therefore represents a unique opportunity to bring new information on the physiological processes under the control of genes on an integrated scale. The potential of this combined approach has been shown recently, with the identification of markers for hepatic lipotoxicity by the global analysis of proteins and lipids in 107 murine lines^8^, as well as the demonstration of significant variations in proteins and metabolites from the same immune pathways in severe forms of COVID-19^9^.

Furthermore, the availability of combined proteomic and metabolomic data is of interest to define multi-omics signatures that may predict more efficiently disease development and progression^10^. As an example, the statistical integration of proteomic, metabolomic and lipidomic data (by concatenating selected variables using a Naïve Bayes classifier, or by fusing Random Forest and Linear Discriminant Analysis models learned from each of the data blocks) has proven to provide a better predictive performance than that obtained with each of the individual type of blocks in the case of type 1 diabetes status^11^.

The interest in integrating proteomic and metabolomic data also stems from the common technology used in both approaches, i.e. liquid chromatography coupled to high-resolution mass spectrometry^12^. At the computational level, common formats exist for raw and pre-processed data, such as mzML^13^ and mzTab^14^, respectively. Computational tools which can process either types of mass spectrometry raw data are already available^15–18^. Moreover, sample preparation protocols for the simultaneous extraction of proteins and metabolites have been proposed^19–21^ enabling to combine both omics within an unique analytical strategy.

While a few studies have reported the relevance of integrating proteomic and metabolomic approaches for obtaining deeper insight into disease development or into the underlying biochemical mechanisms, the routine use of such a combined strategy is not straightforward for the deep phenotyping of large cohorts. Here we address the possibility of generating and making publicly available a dataset combining both approaches in a fully non-targeted, standardized, and reproducible way.

As part of the large-scale characterization of mouse models in which each gene is inactivated^3,22^, we focused on two knock-out mouse models for the *Lat* (linker for activation of T cells; MGI:1342293) and *Mx2* genes (MX dynamin-like GTPase 2; MGI:97244), respectively, as well as the control line (*WT*), generated by the Phenomin-ICS infrastructure, member of the International Mouse Phenotyping Consortium (IMPC). On the one hand, *Lat*, besides its role in T-cell receptor (TCR) signalling^23^, has been shown to be involved in neurodevelopmental diseases^24^. On the other hand, *Mx2* is one of the coding genes in the genome region modelling Down syndrome in mice^25^. The characterization of mouse lines is currently based on a battery of animal phenotypic tests (anatomy, behaviour, histology, haematology, physiology), the results of which feed the IMPC database (https://www.mousephenotype.org). To further characterize these models, global molecular approaches are required^26^. The originality of our study is to provide, in addition to the preclinical data, a comprehensive molecular characterization by proteomic and metabolomic analyses of liver and plasma samples from the *Lat*, *Mx2* and *WT* mouse models.

Our study consists of 9 datasets (1 preclinical, 2 proteomics and 6 metabolomics), generated by the four French infrastructures for mouse phenogenomics, proteomics, metabolomics and bioinformatics, and are publicly available in the *ProMetIS* R package^27^ to ensure accessibility, interoperability, and reusability following the FAIR principles^28^. The dataset provides access to unique molecular functional information on the *Lat* and *Mx2* genes. Furthermore, the protocols and computational workflows provided here can be considered as generics, and as such, they can be easily extended to a larger number of individuals and tissues. In particular, this pilot study paves the way for the inclusion of proteomics and metabolomics analyses in the standardized IMPC pipelines for the characterization of mouse mutants. Finally, the *ProMetIS* resource will be of great interest to develop new bioinformatic and biostatistic methods for the processing and integration of (pre)clinical, proteomic and metabolomic approaches.

## Methods

### Preclinical data

#### Mouse lines

**Fig. 1:**
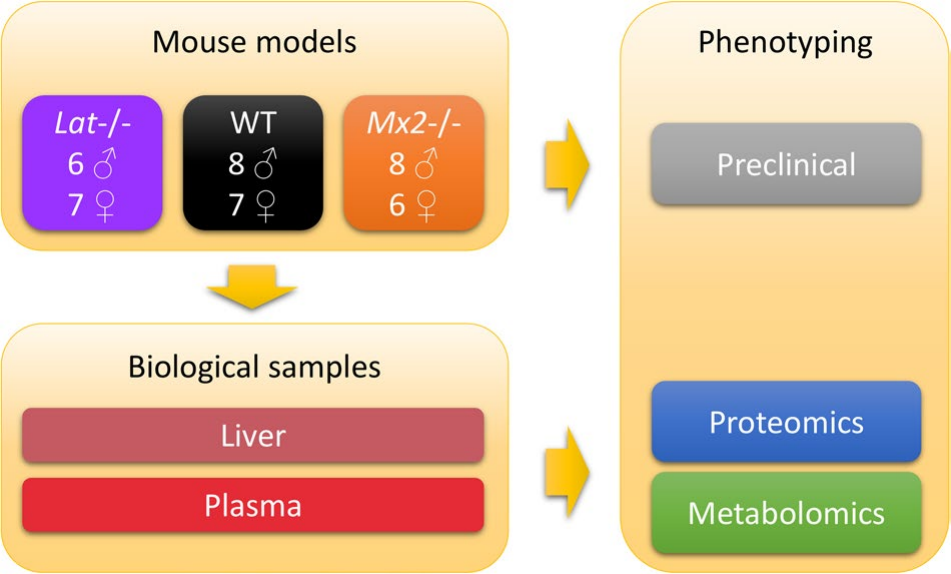
Experimental design. A total of 42 mice from the *Lat*−/− and *Mx2*−/− genotypes (as well as the wild-type controls), and from both sex, were analyzed by a series of phenomic (preclinical) measurements. Liver and plasma samples from all mice were further analyzed at the molecular level by proteomics and metabolomics, except for 6 plasma samples which could be not analyzed by proteomics due to the limited volume available (*Lat*: 1 male and 1 female, *Mx2*: 1 male and 1 female, *WT*: 2 males).

The *Lat*^*em1(IMPC)Ics*^ and *Mx2*^*em1(IMPC)Ics*^ homozygote mouse mutant lines were generated at the Mouse Clinical Institute in Illkirch, France (Phenomin-ICS, http://www.ics-mci.fr), as part of the International Mouse Phenotyping Consortium (IMPC)^29^. Briefly, mice were generated on a pure C57BL/6 N background using CrispR/Cas9. gRNAs were selected with the Crispor program (http://crispor.tefor.net/crispor.py) to delete a critical exon, here exon 2 for both *Lat* and *Mx2* genes, that will introduce a change in the open reading frame and the stop of translation. After microinjection in the pronucleus of C57BL/6 N fertilized eggs, 16 pups were born in the *Lat* study, and 6 of them had the expected deletion of exon 2 of *Lat*. A line was established from one founder (#13), the size of the deletion was 242 bps. Primers F1 (CTTCTTGGTCACGCTCCTGGCTG) and R1 (ATGCTTCTTGGGTACAAACTGGCAG) were used for genotyping (*WT* allele: 600 bps, *KO* allele: 358 bps). For the *Mx2* gene, 15 pups were born after microinjection in C57BL/6 N fertilized eggs and 2 carried the expected deletion of exon 2. A line was established from one founder (#9), the size of the deletion was 411 bps. Primers F1 (TGGAACAGACACCTAAGTCTTGGTC) and R6 (CAGACACCAAGTGGCTTCTCCCAGG) were used for genotyping (*WT* allele: 856 bps, *KO* allele: 445 bps). The two mouse mutant mice were bred to homozygosity in 2 generations on the C57BL/6 N genetic background and maintained in a temperature-controlled facility (20–22 °C) on a 12‐hour light/dark cycle (7AM-7PM) with free access to standard chow diet (D04, SAFE Villemoisson-sur-Orge, France). The homozygous animals, 6 to 8 males and females, from the *Lat* and *Mx2 KO* lines, were obtained from homozygotes breeding at the 3rd generation. Wild-type control individuals were derived from the C57BL/6 N colony maintained under the same conditions (Fig. 1).

#### Sample collection

At the end of the phenotyping pipeline (16 weeks of age), blood was collected by retro orbital puncture under isoflurane anaesthesia for biochemistry and haematology analysis according to the “Clinical Chemistry” protocol available on the IMPReSS database (see the Supplementary File 3). Briefly, 160–200 μl of plasma were collected in a gel tube containing lithium Heparin in the morning. Whole blood samples were centrifuged for 10 minutes at 5,000 g in a refrigerated centrifuge set at 8 °C. The plasma samples were aliquoted and frozen (−20 °C) until transfer to the partners for metabolomic and proteomic analyses. Due to limited volume, 6 plasma samples (*Lat*: 1 male and 1 female, *Mx2*: 1 male and 1 female, *WT*: 2 males) were not analyzed by proteomics. Upon sacrifice, i) all animals were weighted and measured, ii) an extensive necropsy was performed on two mice per sex and genotype, and iii) all organs defined in IMPC protocol were collected on these animals. The liver tissues from all animals were dissected, snap-frozen in liquid N_2_, and transferred to partners for metabolomics and proteomics analysis.

#### Phenotyping

**Fig. 2:**
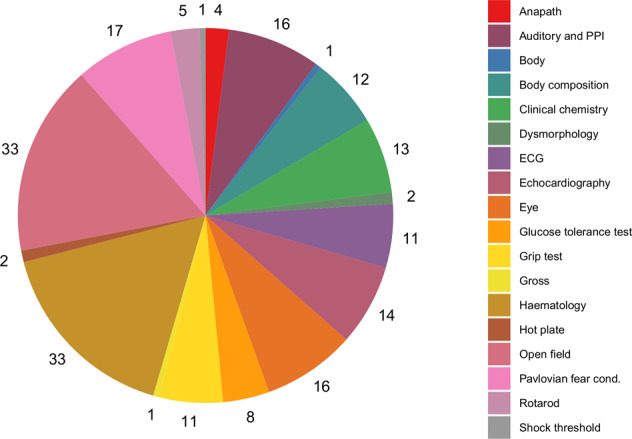
The 200 phenotypic (preclinical) measurements cover a large panel of anatomical, behavioural, histological, haematological, and physiological tests (see the Supplementary File 3 for the identifiers and the links to the experimental procedures in the online IMPReSS database).

Phenotyping data were collected between the age of 4 and 16 weeks (Fig. 2). Both mutant and wild-type mice were tested through a broad-based primary phenotyping pipeline in all the major adult organ systems and most areas of major human diseases. Phenotyping tests are standardized and cross-validated between centres of the consortium^30^, and all procedures used to generate data from mutant and wild-type control mice followed the defined and validated Phenomin-ICS protocols available on the IMPReSS database (https://www.mousephenotype.org/impress; see the Supplementary File 3 for the list of identifiers and links to the specific procedures).

#### Ethical statement

Phenomin-ICS is accredited by the French Ministry of Higher Education, Research and Innovation, and the French Ministry of Agriculture (agreement #A67-218-37), and in accordance with the Directive of the European Parliament: 2010/63/EU, revising/replacing Directive 86/609/EEC and with French Law (Decree n°2013-118 01 and its supporting annexes entered into legislation 01 February 2013) relative with the protection of animals used in scientific experimentation. All animal experiments were approved by local ethical committees: Approval Committee: Com’Eth N°17 and French Ministry of Higher Education, Research and Innovation (Approval license: MESR: APAFIS#4789-2016040511578546). Animal studies were supervised in compliance with the European Community guidelines for laboratory animal care and use. Every effort was made to minimize the number of animals used and their suffering.

### Proteomics

#### Sample preparation

Mice liver proteins were extracted in 10% w/v Laemmli buffer (Tris 10 mM pH 6.8, EDTA 1 mM, beta-mercaptoethanol 5%, SDS 5%, glycerol 10%) with 1% v/v protease inhibitors (Pierce #P8340), firstly under gentle agitation at room temperature for 2 hours, and then using quick sonication on ice (3 × 10 s, 135 W) to ensure complete solubilisation. Samples were centrifuged at 10,000 g for 10 min to remove possible cell debris. Protein concentrations in the supernatants were determined using the RC-DC Protein Assay (BioRad, Hercules, CA, USA). Then 1% blue bromophenol was added, the samples were heated at 95 °C for 5 min, 50 µg of sample were loaded on an in-house prepared 4% acrylamide SDS-PAGE stacking gel and run at 50 V for 13 min. Proteins were fixed and then stained using colloidal Coomassie blue (Fluka, Buchs, Switzerland) for 5 min. The stacking bands were manually excised, destained, reduced for 1 hour with 10 mM dithiothreitol, and alkylated for 20 min with 55 mM iodoacetamide in the dark. Overnight digestion was then performed at 37 °C using 1:50 enzyme:protein ratio of Trypsin/Lys-C Mix (#V507A, Promega, Madison, USA). Peptides were extracted during 1 hour with 160 µl acetonitrile (ACN). Organic solvent was eliminated using a vacuum centrifuge (SpeedVac, Savant, Thermo scientific, Waltham, MA, USA), and peptides were re-suspended in 140 µl of water acidified with 0.1% formic acid (FA).

For analysis of plasma proteins, filter-aided sample preparation was performed using Amicon Ultra devices (0.5 mL, cutoff 10 kDa, Merck Millipore). Three microliters of each plasma sample were diluted with 350 µl of 4 M urea, 50 mM ammonium bicarbonate buffer and centrifuged in the device at 14,000 g and 4 °C, to a concentrated volume of 50 µl. For reduction of cysteine residues, 350 µl of 10 mM TCEP in 4 M urea, 50 mM ammonium bicarbonate buffer were added on the device, and the samples were incubated 10 min at room temperature. Sample volume was reduced again down to 50 µl by centrifugation at 14,000 g and 4 °C. Alkylation of cysteine residues was performed by addition of 350 µl of 55 mM iodoacetamide in 4 M urea, 50 mM ammonium bicarbonate buffer, followed by 15 min incubation in the dark, and concentration of the samples by centrifugation of the filter device. Two additional washes of the samples were performed with 4 M urea, 50 mM ammonium bicarbonate buffer. Proteins were digested with 1:30 enzyme:protein ratio of Trypsin/Lys-C Mix (Promega) in 4 M urea, 50 mM ammonium bicarbonate, for 2 h at 37 °C. The concentration of urea was then reduced to 1 M by dilution with 50 mM ammonium bicarbonate, and the samples were further incubated for 3 h at 37 °C. Resulting peptides were collected by centrifugation of the 10 kDa filter, which was further rinsed with 100 µl of 0.5 M NaCl. Peptides were desalted on C18 spin columns (Pierce), dried in a vacuum centrifuge, and re-suspended in 180 µl of water containing 2% ACN and 0.1% trifluoroacetic acid (TFA).

Before LC-MS analysis, a set of reference peptides (iRT kit; Biognosys AG, Schlieren, Switzerland) was added to all samples. Furthermore, for the quality control of the mass spectrometry analysis sequence, a pooled quality control (QC) sample was constituted by pooling 1 µl of each sample, either for the liver tissue samples or for the plasma samples series. The samples were stored at 4 °C and analyzed within a week.

#### Analytical chemistry

NanoLC-MS/MS analyses of the tryptic peptides obtained from liver proteins were performed on a nano-UPLC system (nanoAcquityUPLC, Waters, USA) coupled to a quadrupole-Orbitrap hybrid mass spectrometer (Q-Exactive plus, Thermo Scientific, San Jose, CA). Briefly, 1 µl (320 ng) of each sample was concentrated/desalted on a trap column (Symmetry C18, 180 μm × 20 mm, 5 µm; Waters) using 99% of solvent A (0.1% FA in water)/1% solvent B (0.1% FA in ACN) at a flow rate of 5 µl/min for 3 minutes. Afterwards, peptides were eluted from the separation column (BEH130 C18, 75 μm × 250 mm, 1.7 μm; Waters) maintained at 60 °C using a 79 min gradient from 1–35% of B at a flow rate of 450 nL/min. The Q-Exactive Plus was operated in positive ion mode with source temperature set to 250 °C and spray voltage to 1.8 kV. The mass spectrometer was operated in data-dependent acquisition mode, and spectra were acquired through automatic switching between full MS and MS/MS scans. Full scan MS spectra (300–1800 m/z) were acquired at a resolution of 70,000 at m/z 200 with an automatic gain control (AGC) value set to 3 × 10^6^ ions, a maximum injection time set to 50 ms, and the lock-mass option enabled (polysiloxane, 445.12002 m/z). Up to 10 most intense multi-charged precursors per full MS scan were isolated using a 2 m/z window and fragmented using higher energy collisional dissociation (HCD, normalized collision energy of 27 eV). MS/MS spectra were acquired at a resolution of 17,000 at m/z 200 with an AGC value set to 1 × 10^5^, a maximum injection time set to 100 ms, and the peptide match selection option was turned on. Dynamic exclusion of already fragmented precursors was set to 60 seconds. The system was fully controlled by the Xcalibur software (v3.1.66.10; Thermo Fisher Scientific). MS data were saved in .raw file format (Thermo Fisher Scientific) using XCalibur.

For analysis of plasma samples, peptides were separated using a nano-UPLC system (Ultimate NCS-3500RS System; Thermo Fisher Scientific) coupled to a quadrupole-Orbitrap hybrid mass spectrometer (Q-Exactive HFX, Thermo Scientific, San Jose, CA). Five microliters of each sample were loaded on a C18 precolumn (300 µm inner diameter × 5 mm, Thermo Fisher Scientific) in a solvent made of 2% ACN and 0.05% TFA, at a flow rate of 20 µl/min. After 3 min of desalting, the precolumn was switched online with the analytical C18 column (75 µm inner diameter × 50 cm, Acclaim PepMap C18, 2 µM, Thermo Fisher Scientific), equilibrated in 90% solvent A (5% ACN, 0.2% FA) and 10% solvent B (80% ACN, 0.2% FA), in order to speed-up the elution of the peptides at the beginning of the analytical run. Peptides were then eluted by a gradient composed of 2 slopes (from 8 to 24% of ACN during 50 min, and from 24% to 36% of ACN during 10 min), at a flow rate of 350 nl/min. Full scan MS spectra (350–1400 m/z) were acquired with a resolution of 60,000 and an AGC target of 3 × 10^6^ ions. The 6 most intense ions were selected (1.3 m/z window) for fragmentation by high energy collision induced dissociation (normalized collision energy of 28 eV), and the resulting fragments were analysed at a resolution of 30000, using an AGC target of 1 × 10^5^ and a maximum fill time of 54 ms. Dynamic exclusion was used within 30 s to prevent repetitive selection of the same peptide.

All sample analyses, for either the liver or plasma series, were randomly ordered (considering the genotype, sex and weight of the mice). Pooled QCs were injected every 5 samples. To minimize carry-over, one solvent blank injection was included between all samples.

#### Data processing

Spectrum identification: Raw files were converted to.mgf peaklists using MsConvert and were submitted to Mascot database searches (version 2.5.1, MatrixScience, London, UK) against a murine protein sequences database downloaded from the SwissProt website (2018_08_20), to which common contaminants, iRT and decoy sequences were added. The concatenated database contains 2 × 17 111 protein entries. Spectra were searched with a mass tolerance of 5 ppm in MS mode and 0.07 Da in MS/MS mode. Two trypsin missed cleavages were tolerated. Carbamidomethylation of cysteine residues was set as a fixed modification. Oxidation of methionine residues and acetylation of proteins n-termini were set as variable modifications. Identification results were imported into the Proline software (http://proline.profiproteomics.fr)^31^ for validation. Peptide Spectrum Matches (PSM) with pretty rank equal to one were retained. False Discovery Rate was then optimized to be below 1% at PSM level using Mascot Adjusted E-value and below 1% at Protein Level using Mascot Mudpit score. Label Free Quantification: Peptides Abundances were extracted thanks to the Proline software^31^ version 1.6 using an m/z tolerance of 5 ppm. Alignment of the LC-MS runs was performed using Loess smoothing. Cross assignment of peptide ions abundances was performed among the samples and controls using an m/z tolerance of 5 ppm and a retention time tolerance of 42 s. Protein abundances were computed using the median ratio fitting of the unique peptides abundances normalized at the peptide level using the median.

#### Statistical post-processing

Protein Abundances were loaded into the *ProStaR* software version 1.16 (http://www.prostar-proteomics.org)^32^, log2 transformed and associated to their conditions (*WT*, *Lat* or *Mx2*). Proteins with at least 80% of non-missing values in at least one condition of the one vs. one comparison were kept for further statistical analysis. Contaminants and reverse hits were filtered out. Residual missing values were imputed according to their nature^33^: partially observed values were imputed according to the measured values (Structured Least Squares Algorithm)^34^, whereas values missing in the entire condition were imputed in a conservative way (approximation of the lower limit of quantification by the 2.5% lower quantile of each replicate intensity distribution).

The mass spectrometry proteomics data have been deposited to the ProteomeXchange Consortium via the PRIDE partner repository^35^: the liver dataset is available with the dataset identifier PXD028416^36^ and the plasma dataset is available with the dataset identifier PXD028550^37^.

### Metabolomics

Three complementary untargeted metabolomics methods based on Ultra-High Performance Liquid Chromatography coupled to High-Resolution Mass Spectrometry (which we will abbreviate as LC-MS in the following for simplicity) were used to ensure a good metabolome coverage. Acquisition of plasma metabolomics data was performed on the three analytical platforms, while metabolomics data from mouse liver was obtained only on two platforms (HILIC and Hypersil C18 as named below), mainly due to the limited sample amounts.

#### Sample preparation

*for further analysis under HILIC and Hypersil C18 conditions*: mouse plasma metabolite extraction was performed twice from 50 µl of plasma following methanol-assisted protein precipitation as previously described^38^. Briefly, a volume of 200 μl of methanol containing internal standards at 3.75 µg/ml (Dimetridazole, 2-amino-3-(3-hydroxy-5-methyl-isoxazol-4-yl)propanoic acid (AMPA), 2-methyl-4-chlorophenoxyacetic acid (MCPA), Dinoseb (Sigma-Aldrich, Saint-Quentin Fallavier, France) was added to the 50 µl of plasma. The resulting samples were then left on ice for 90 min until complete protein precipitation. After a centrifugation step at 20,000 g for 15 min at 4 °C, supernatants were collected and dried under a nitrogen stream using a TurboVap instrument (Thermo Fisher Scientific, Courtaboeuf, France) and stored at −80 °C until analysis. Prior to LC-MS analysis, dried extracts were resuspended in 150 µl of 10 mM ammonium carbonate (pH 10.5) and ACN (40:60, v/v) containing the external standards (mixture of 13C-glucose and 15N-aspartate at 200 µg/ml, ethylmalonic acid at 30 µg/ml, amiloride at 100 µg/ml, prednisone, atropine sulfate and metformin at 10 µg/ml, colchicine and imipramine at 5 µg/ml) for ZIC-pHILIC analysis or water/ACN (95:5, v/v), containing 0.1% FA and the external standards for C18 analysis. A pooled QC sample was obtained by pooling 20 µl of each sample preparation. Aliquots of this QC sample were injected every 5 samples to evaluate potential signal drift of any metabolite, while the external standards added to all samples were used to check for consistency of signal and retention time stability throughout the experiments.

Mouse liver metabolites extraction was performed from ~25 mg of tissue. Samples were resuspended in 170 µl of ultrapure water, and then sonicated 5 times for 10 s using a sonication probe (Vibra Cell, Bioblock Scientific, Illkirch, France). At this step, 20 µl of each sample were withdrawn for further determining the total protein concentration (colorimetric quantification/Pierce BCA Protein Assay Kit, Thermo Fisher Scientific, Courtaboeuf, France). A volume of 350 µl of methanol was added to the remaining 150 µl of tissue lysate. Cell debris were then removed by centrifugation for 15 min at 4 °C and 20,000 g. The resulting samples were then left on ice for 90 min until complete protein precipitation. After a final centrifugation step at 20,000 g for 15 min at 4 °C, supernatants were recovered and split into two equal aliquots for C18 and HILIC analyses. Resulting aliquots were then dried under a stream of nitrogen using a TurboVap instrument (Thermo Fisher Scientific, Courtaboeuf, France) and stored at −80 °C until analysis. Prior to LC-MS analysis, dried extracts were resuspended to reach a fixed protein concentration (equivalent to 20 mg/mL) as described above for plasma-derived metabolites using mobile phase mixtures containing external standards (internal standards were not added for liver metabolomics). After reconstitution, the tubes were vortexed and incubated in an ultrasonic bath for 5 min and then centrifuged at 20,000 g for 15 min at 4 °C. Supernatant was transferred into 0.2 ml vials. QC samples were prepared and analysed as described above for plasma samples.

#### Sample preparation

*for further analysis under HSS T3 C18 conditions*: 100 µl of mouse plasma were extracted as follows: samples were slowly thawed on ice at room temperature and protein precipitation was performed by addition of 150 µl of ice-cold methanol. This mixture was vortexed and placed at −20 °C for 30 min. After a 10 min centrifugation (4 °C, 15493 g, Sigma 3-16PK, Fischer Bioblok Scientific), the supernatant was divided into two aliquots, dried completely (EZ2.3 Genevac, Biopharma Technologies France) and stored at −80 °C until further analysis. Just before analysis, 200 µl of injection solvents (water and ACN 50/50 + 0.1% FA) was added to the dried fractions. A pooled QC sample was prepared by mixing 5 µl from each extracted sample and injected every 6 samples.

#### LC-HRMS Analysis

*HILIC and Hypersil C18 analyses*: The ultra-high performance liquid chromatographic (UHPLC) separation was performed on a Hypersil GOLD C18 1.9 µm, 2.1 mm × 150 mm column (RP) at 30 °C (Thermo Fisher Scientific, les Ulis, France), and on a Sequant ZICpHILIC 5 µm, 2.1 × 150 mm (HILIC) at 15 °C (Merck, Darmstadt, Germany). All chromatographic systems were equipped with an on-line prefilter (Thermo Fisher Scientific, Courtaboeuf, France). Experimental settings for each LC/MS condition are described below. Mobile phases for the C18 column were 100% water in A and 100% ACN in B, both containing 0.1% FA. Regarding HILIC, phase A consisted of an aqueous buffer of 10 mM ammonium carbonate in water adjusted to pH 10.5 with ammonium hydroxide, whereas pure ACN was used as solvent B. Chromatographic elutions were achieved under gradient conditions as follows: (i) C18-based system: the flow rate was set at 500 µl/min. The elution consisted of an isocratic step of 2 min at 5% phase B, followed by a linear gradient from 5 to 100% of phase B for the next 11 min. These proportions were kept constant for 12.5 min before returning to 5% B for 4.5 min; (ii) HILIC-based system: the flow rate was 200 µl/min. Elution started with an isocratic step of 2 min at 80% B, followed by a linear gradient from 80 to 40% of phase B from 2 to 12 min. The chromatographic system was then rinsed for 5 min at 0% B, and the run ended with an equilibration step of 15 min (80% B).

LC-MS analyses were performed using a U3000 liquid chromatography system coupled to a first generation Exactive mass spectrometer from Thermo Fisher Scientific (Courtaboeuf, France) fitted with an electrospray ionization (ESI) source operated in the positive and negative ion modes. The software interface was Xcalibur (version 2.1; Thermo Fisher Scientific, Courtaboeuf, France). The mass spectrometer was calibrated before each analysis in both ESI polarities using the manufacturer’s predefined methods and recommended calibration mixture provided by the manufacturer (external calibration). The Exactive mass spectrometer was operated with capillary voltage at −3 kV in the negative ionization mode and 5 kV in the positive ionization mode and a capillary temperature set at 280 °C. The sheath gas pressure and the auxiliary gas pressure were set, respectively, at 60 and 10 arbitrary units with nitrogen gas. The mass resolution power of the analyzer was 50,000 (full width at half maximum) at *m/z* 200, for singly charged ions. The detection was achieved from *m/z* 85 to 1000 for RP conditions in the positive ionization mode and from *m/z* 75 to 1000 for HILIC conditions in the negative ionization mode.

#### LC-HRMS analysis

*Acquity HSS T3 C18 analyses*: To retain very polar compounds while keeping the elution of less polar metabolites, an HSS T3 column was selected as complementary method^39^, as this bonding process utilizes a trifunctional C18-alkyl phase bonded at an intermediate 1.6 µmol/m2 ligand density to promote highly polar compounds retention, without extensive retention of less polar components. Metabolic profiles were determined using an U3000 liquid chromatography system (Thermo Fisher Scientific, San Jose, CA, USA) coupled to a high-resolution Bruker Impact ll UHR-QTOF (Bruker Daltonics, Wissembourg, France) equipped with an ESI source. Chromatographic separation was performed on a Waters HSS T3 column (150 × 2.1 mm, 1.8 µm) at 0.4 ml/min, 30 °C and using an injection volume of 5 µl. Mobile phases A and B were water and ACN with 0.1% FA, respectively. The elution gradient was 0% B (2 min), 0–100% B (13 min), 100% B (7 min), 100-0% B (0.1 min) and 0% B (3.9 min for re-equilibration). The mass resolution of the mass spectrometer was 50,000 and mass accuracy ranged from 0.8–2 ppm. Samples were analyzed in the positive and negative ionisation modes (Pos, Neg). Capillary and end plate offset voltages were set at 2,500 V and 500 V for the ESI source. The drying gas temperature was 200 °C and nebulisation gas flow was 10 l/min. Mass spectrum data was acquired in full-scan mode over mass range 50–1000 mass-to-charge ratio (*m/z*).

The orders of injection of the samples into the LC-HRMS instruments were randomized to ensure that there was no distribution differences between genotypes and between sexes.

#### Data processing

Raw files were converted to the mzML or mzXML format with the ProteoWizard software^40^. mzML files were further compressed by using the MS-Numpress tool^41^. Mass spectra were processed using the *xcms* R package^42^ deployed in the Workflow4Metabolomics Galaxy platform (https://workflow4metabolomics.org)^43^. In particular, the peak detection and quantification was performed with the *centWave* algorithm^44^ by using a value of 10 ppm and peak width values of 10,40 (Hypersil C18), 15,90 (HILIC) and 5,20 (Acquity HSS T3 C18). Annotation of the chemical redundancy (adducts, isotopes, fragments) was performed with the CAMERA software^45^.

#### Statistical post-processing

**Fig. 3:**
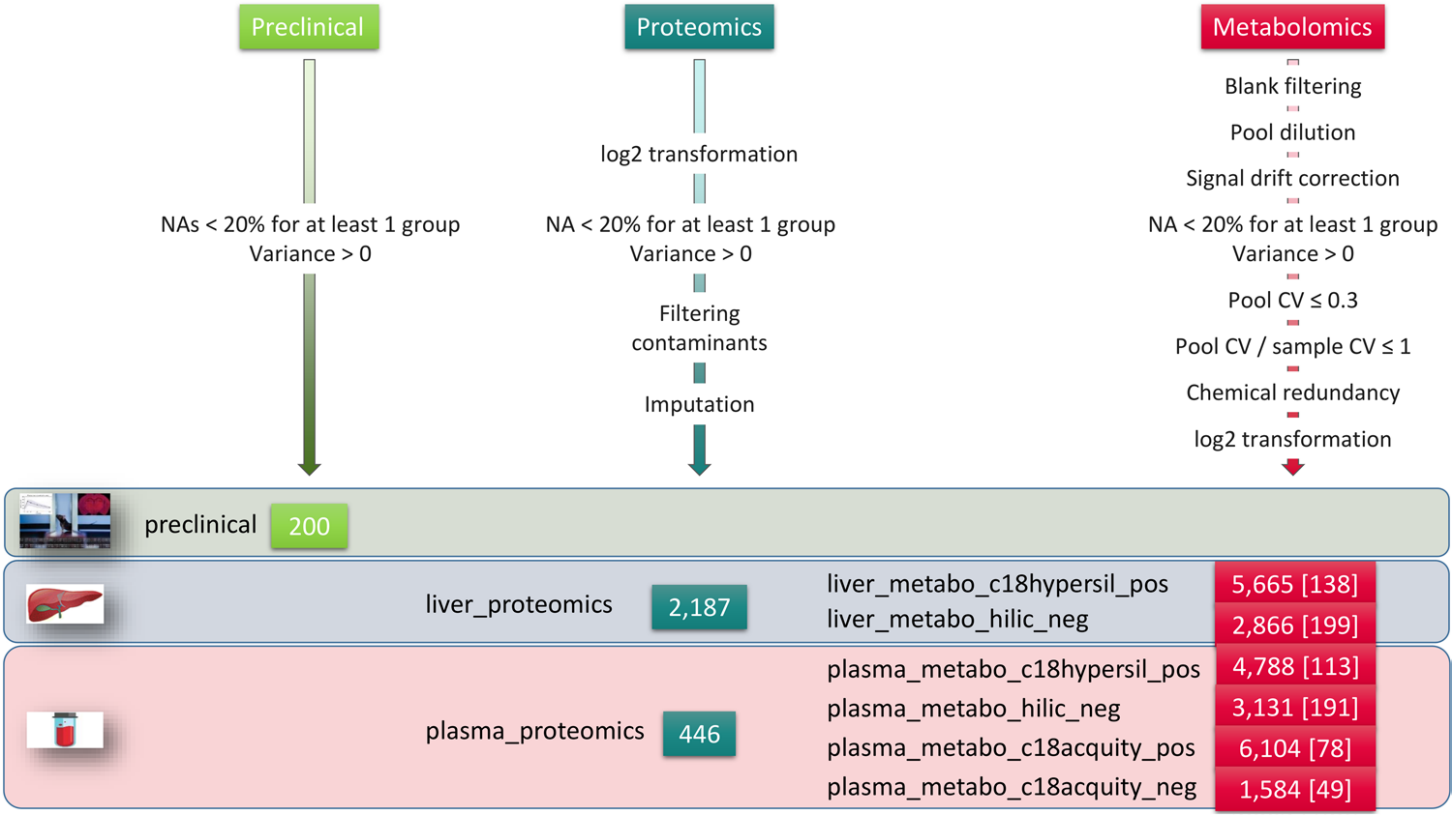
Post-processing of the *ProMetIS* datasets. The number of features in each of the 9 datasets is shown. The number of annotated metabolites is indicated in squared brackets. The names of the metabolomics datasets include the reference to the chromatographic column (c18hypersil: Hypersil GOLD C18, hilic: ZIC-pHILIC, and c18acquity: Acquity HSS-T3) and the ionization mode (pos: positive, and neg: negative).

**Fig. 4:**
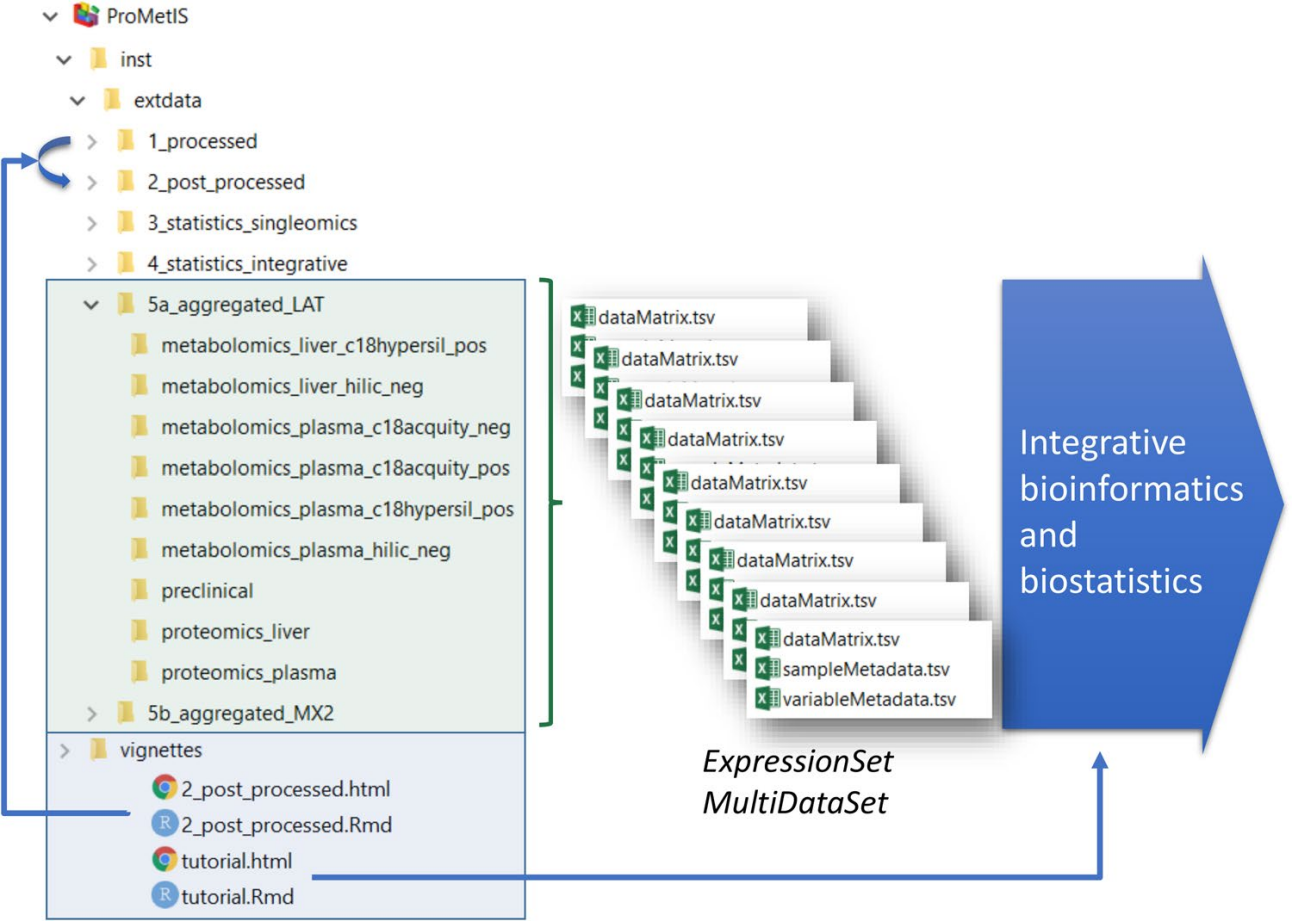
Data and code availability in the *ProMetIS* package^27^. The data are available in the *extdata* folder, which is organized into subfolders corresponding to the successive steps of the analysis (e.g., *1_processed*, *2_post_processed*, *5_aggregated*, etc.). Within each subfolder, the 9 datasets are stored as a triplet of tabular files containing the matrix of intensities (dataMatrix.tsv), the sample metadata (sampleMetadata.tsv) and the variable metadata (variableMetadata.tsv). For each step, a vignette describes the methods used to process the data. As an example, the *2_post_processed* vignette details how the datasets were post-processed, between the *1_processed* and the *2_post_processed* folders (note that only the post-processing of the metabolomics files is described, since the proteomics files were post-processed separately with the *ProStaR* software). The package thus provides a full access to both data and code, to ensure reproducibility of the results. The *tutorial* vignette describes how to access the final *aggregated* data to perform integrative bioinformatics and biostatistics analyses.

Variables whose mean intensity in the biological samples was less than 3 times that of the blanks were discarded. The inclusion of diluted pooled QCs in the Orbitrap datasets enabled further filtering of variables whose linear (Pearson) correlation between the intensity and the inverse of the dilution factor was lower than 0.7. The signal drift observed in the plasma datasets was estimated for each variable by locally weighted (*loess*) regression and corrected^46,47^. The reference samples for the regression were the pooled QC samples for the Hypersil C18/HILIC datasets, and the biological samples for the Acquity HSS T3 datasets: for the latter datasets, the normalization based on the pooled QCs was shown to provide only limited correction of the signal drift (as assessed both visually and by the Drift PCA metric described in the Technical Validation section), and the biological samples were therefore used as the reference instead^48,49^. Variables with a coefficient of variation in the QCs above 30% or greater than the coefficient of variation in the samples were then filtered out. Chemically redundant features (i.e. isotopes, adducts, fragments) were discarded based on the conjunction of three criteria^50^: Pearson correlation of sample profiles above 0.9, difference between retention times below 6 s, and m/z difference matching a reference list corresponding to isotopes, adducts and fragments at a 0.005 Da tolerance. Finally, intensities were log2 transformed. All steps of this post-processing workflow (Fig. 3) are described in the *2_post_processed* vignette from the *ProMetIS* package^27^ (Fig. 4), which relies on methods from the *phenomis* R package (https://github.com/SciDoPhenIA/phenomis).

#### Annotation

Metabolite features were annotated according to accurately measured masses and chromatographic retention times by using a local spectral database containing the reference spectra of more than 2,000 authentic standard compounds analyzed in the same analytical conditions, and providing a comprehensive spectral information (i.e. protonated or deprotonated molecules, adducts and in-source fragment ions)^38,51^. To be identified, ions had to match at least two orthogonal criteria (e.g., accurately measured mass and retention time) to those of an authentic chemical standard analyzed under the same analytical conditions, as proposed by the Metabolomics Standards Initiative^52^. Additionally, metabolite annotations from the Acquity HSS T3 platform were confirmed by running LC-MS/MS experiments, conducted on the same QC samples, and with the instrument set in the targeted (Multiple Ion Monitoring) acquisition mode. Resulting MS/MS spectra were then manually matched to those included in the in-house spectral database and acquired using different collision energies. For more intelligibility, the annotation confidence was detailed in the datasets regarding the decision criteria (a: accurate mass; b: retention time; c: consistent MS/MS; d: consistent MS/MS with external database).

## Data Records

### Sample metadata

Throughout the project (including in the data repositories and in the *ProMetIS* package, the sample names of the 42 mice have been abbreviated to facilitate data manipulation. In particular, a unique project identifier composed of 3 digits is used (ranging from 501 to 862). This identifier is preceded by an uppercase letter referring to the genotype (L = *Lat*, X = *Mx2*, W = *WT*) and followed by a lowercase letter referring to the sex (m = male, f = female): For example, the ‘X501f’ sample comes from the female mouse #501 carrying the *Mx2* deletion.

The correspondence between the project identifiers and the reference IDs from the IMPC database is available in the *ProMetIS* package (in the *preclinical* subfolders). This table also contains the links to the phenotyping data on the IMPC database. A copy of the table has been included in the Supplementary File 2 for the reader’s convenience (*sampleMetadata* sheet).

### Raw and processed data

Preclinical data are available on the IMPC database at https://www.mousephenotype.org/data/genes/MGI:1342293^53^ and https://www.mousephenotype.org/data/genes/MGI:97244^54^. MS proteomics data (raw, processed and post-processed files), including peptide data and reference files (readme, search database,.dat files), form a complete submission in the ProteomeXchange repository^55^. Data were submitted via the PRIDE partner repository under dataset identifiers PXD028416 (liver)^36^ and PXD028550 (plasma)^37^. MS metabolomics datasets (raw and processed files) are available for both matrices on the MetaboLights repository^56^ under the MTBLS1903 identifier^57^.

### Post-processed data

Each post-processed dataset is publicly available in the *ProMetIS* R package^27^, as 3 tabular files containing the intensities (dataMatrix), the sample metadata (sampleMetadata) and the variable metadata (variableMetadata) in the *extdata* folder, either in the *2_post_processed* subfolder or in the *5a/b_aggregated_LAT/MX2* subfolders (the latter further include results from statistical analyzes as additional sample and variable metadata). These files may be conveniently opened with any spreadsheet editor. They may be imported as an *ExpressionSet*^58^ or *MultipleDataSet*^59^ for further single- or multi-omics analysis in R (see for instance the *tutorial* vignette from the *ProMetIS* package). Alternatively, they may be analyzed by using the Galaxy tools from the Workflow4Metabolomics platform^43,60^. Importantly, the post-processing steps used to generate the metabolomics files are described in the *2_post_processed* vignette from the *ProMetIS* package to meet the Findable, Accessible, Interoperable and Reusable FAIR guidelines^28^. Those post-processed datasets (annotated variables only) are also provided in the Supplementary File 2 for the reader’s convenience (with one sheet per dataset).

## Technical Validation

The *ProMetIS* dataset was generated by the four French Infrastructures for Mouse Phenogenomics (Phenomin-ICS; http://www.phenomin.fr/en-us), Proteomics (ProFI; http://www.profiproteomics.fr), Metabolomics and Fluxomics (MetaboHUB; https://www.metabohub.fr/home.html) and Bioinformatics (IFB; https://www.france-bioinformatique.fr) which meet the international standards for optimal quality, reproducibility and accessibility of the data.

### Preclinical data

Routine procedures to optimize the quality of phenotyping data at the Phenomin-ICS infrastructure include i) standardized pipelines and experiments, ii) automated flagging of outlier variables by comparing the *WT* mice from the project to all other *WT* mice from the same genetic background previously generated by the platform (as described below), and iii) manager data review and validation.

For each quantitative variable, a reference range encompassing 95% of the values from all *WT* male and female mice with the same genetic background in the Phenomin-ICS database is computed (rr95). For the variables which follow a normal distribution (either directly or after an inverse, logarithm or square root transformation), as assessed with the Shapiro-Wilk test, the rr95 is set to the average ± 2 × standard deviation (this is the case for about two thirds of the variables). For the other variables, the rr95 bounds are defined as the 2.5th and 97.5th percentiles of the values. In all cases, a minimal number of 120 animals is recommended by the EP28 standard from the Clinical and Laboratory Standards Institute (CLSI; https://clsi.org) to ensure stable lower and upper reference limits. Variables with an average value in the *WT* mice of the project falling out of the rr95 for males or females are discarded.

**Fig. 5:**
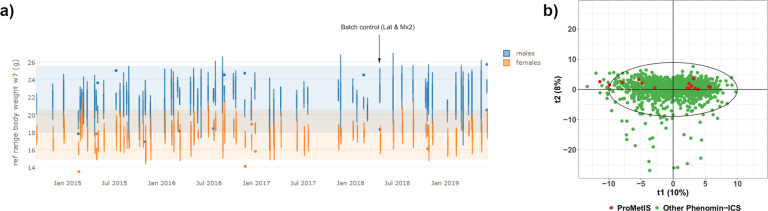
Quality control of the preclinical dataset assessed on the *WT* mice. (**a**) Example of the values of the parameter “body weight at week 7” in the *WT* mice from the *ProMetIS* dataset compared to the other Phenomin-ICS *WT* mice. Blue (respectively, orange) rectangles correspond to the rr95 reference range for males (respectively, females). The batch from the 15 *WT* mice from the *ProMetIS* study is indicated by an arrow. (**b**) Principal component analysis of all *WT* mice from the Phenomin-ICS database. The mice from the *ProMetIS* dataset are colored in red.

Here, the rr95 was computed from 1,112 *WT* mice (573 males and 539 females). The transformation applied to compute the rr95 has been included in the metadata. For all 200 preclinical variables, the 15 *WT* mice from the *ProMetIS* project had average values within the rr95 in males and females, thus confirming the good stability and reproducibility of the measures performed in this study (Fig. 5a). The quality of the preclinical phenotyping was also assessed at the multivariate level by Principal Component Analysis: as expected, *ProMetIS* mice were located within the cloud of all Phenomin-ICS mice on the score plot (Fig. 5b).

### Proteomics data

**Fig. 6:**
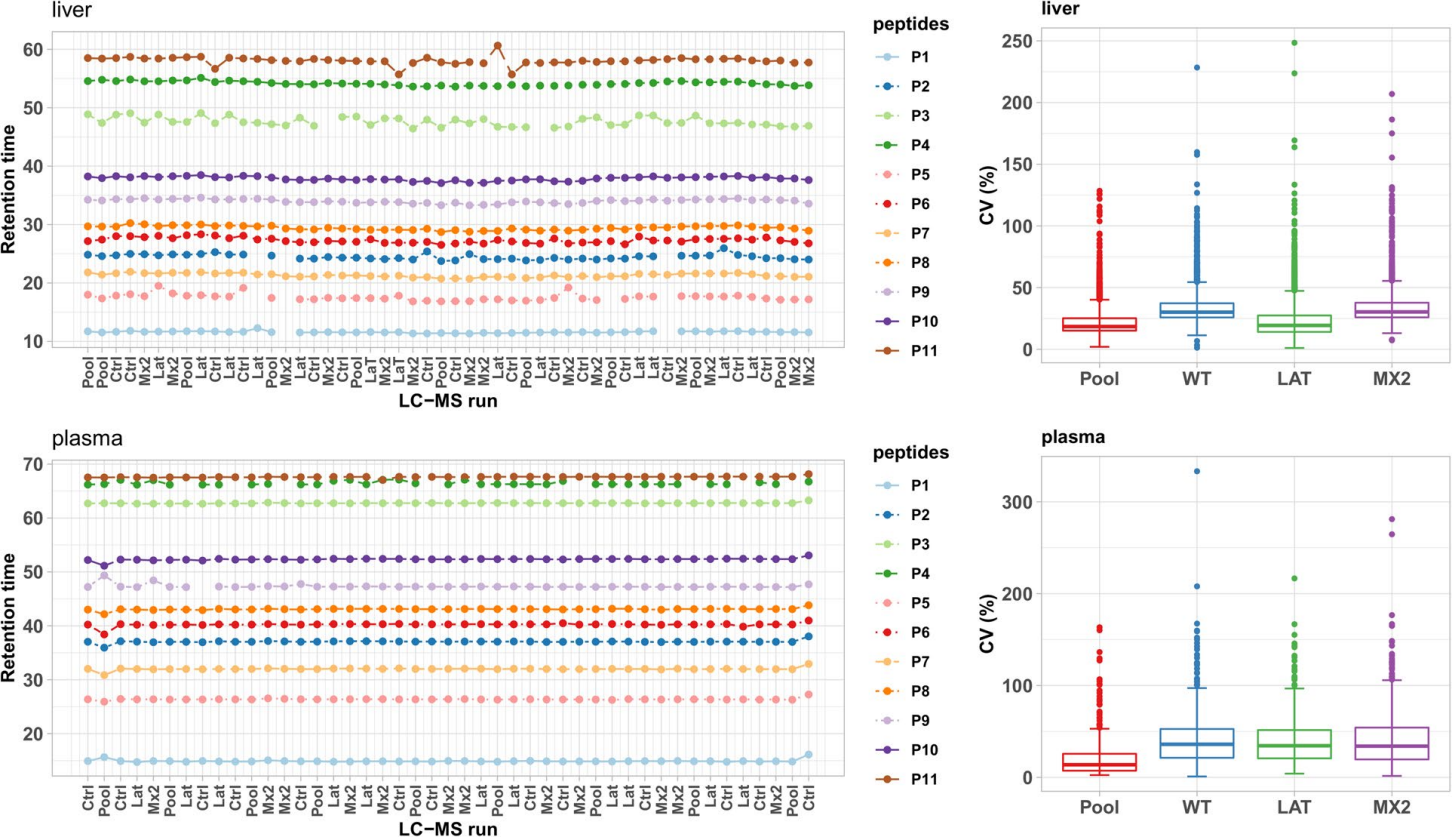
Quality control of the LC-MS proteomics analytical sequences in the liver (top) and plasma (bottom). Left: An internal standard consisting of 11 iRT peptides (iRT kit; Biognosys) was spiked in all samples. Retention times of the 11 spiked in peptides from the iRT kit (Biognosys) are plotted in the chronological order of data acquisition. Mean standard deviation across all liver (respectively, plasma) samples injections are 27 s (respectively, 13.5 s). The sequences of the peptides are: P1: LGGNEQVTR, P2: YILAGVENSK, P3: GTFIIDPGGVIR, P4: GTFIIDPAAVIR, P5: GAGSSEPVTGLDAK, P6: TPVISGGPYEYR, P7: VEATFGVDESNAK, P8: TPVITGAPYEYR, P9: DGLDAASYYAPVR, P10: ADVTPADFSEWSK, P11: LFLQFGAQGSPFLK. Right: CVs of the raw protein intensities per sample type.

The proteomics study of mice liver (respectively, plasma) samples based on tryptic peptides analyzed by nanoLC-MS resulted in the raw quantification of 21,809 (respectively, 6,501) peptides and 2,468 (respectively, 621) proteins at the end of the data processing by the Proline software. Three types of quality control strategies were used throughout the analytical sequence. Analytical conditions were optimised for each of the two tissues since the study was designed for the comparison of mutants vs wild-type samples within each tissue, taken individually. The present experimental design does not therefore allow the direct comparison of protein absolute quantification between the plasma and liver contents. First, an internal standard was spiked in equal amounts in each biological sample and to check the stability of the LC and MS systems. This verification was done post-acquisition of the sequence, through extraction of the retention times (RT) and the raw intensity data associated with the spiked peptides from the quantitative output of the Proline software (Figure S1 of the Supplementary File 1). This spiked-in standard is composed of eleven pure synthetic peptides (iRT kit, Biognosys, # Ki-3002-1). Figure 6 (left) and Figure S1 of the Supplementary File 1 illustrate the reproducibility of the features (retention time and raw intensities, respectively) associated with these iRT peptides across the LC-MS runs of the dataset. In particular, the median CV of intensities across all liver (respectively, plasma) samples injections was 40% (respectively, 31%; Figure S1 of the Supplementary File 1). Second, an external standard of close composition to that of the biological samples themselves was regularly injected during the acquisition sequence. It was prepared by pooling a small aliquot of all initial samples. An LC-MS run of this pool standard was acquired every 4 or 5 analytical runs during the sequence (n = 10 pools for liver samples, n = 8 pools for plasma samples). These runs were included, together with those of the samples, in the data processing performed with the Proline software. The identification metrics extracted from the data of this pooled external standard (i.e. number of proteins, peptides, MS/MS scans; Figure S2, left, and Figure S3, left, of the Supplementary File 1, as well as the CVs of the raw protein intensities, Fig. 6, right, and the correlation between the samples, Figure S4 of the Supplementary File 1) show high repeatability of the results. Third, quality control of the biological datasets was assessed by plotting the same identification and quantitative metrics across all biological samples (Figure S2, right, Figure S3, right, of the Supplementary File 1, and Fig. 6, right). No outlier was identified in the cohorts. The variability of these metrics was close to the one of the pools and was used to assess the global quality of the datasets, excluding any variability related to the composition of the biological samples.

### Metabolomics data

The quality of the metabolomics datasets was investigated after the post-processing step (including the signal drift correction but not the final feature filtering on the pooled QC CVs), as a validation of the data before future statistical studies.

The stability of the signal detection and quantification along the sequence of injection was first assessed on the metabolite internal and external standards which have been spiked in all crude plasma samples before metabolite extraction (internal standards), or in the final extracts just before injection into the LC-HRMS (external standards)^38,61,62^. Of note, the mixture of 13 external standards is used as an “universal” standard solution in the laboratory, i.e. whatever the sample to be analyzed (e.g., biofluid, tissue, bacteria, or fecal samples) and LC-MS conditions, which implies that not all the chemicals are efficiently detected and thus quantified in every type of metabolite extract or analytical conditions (e.g., positive vs negative ionisation). For instance, 9 and 4 external standards were detected in plasma extracts analyzed under *c18hypersil_pos* and *hilic_neg* conditions, respectively. Although all those standard compounds proved efficiently detected in liver extracts when analyzed under *hilic_neg* conditions, *c18hypersil_pos* analysis detected 7 among the 9 external standards potentially due to stronger matrix effects occurring with liver extracts. For all detected standards, the average coefficient of variation was 2% (standard deviation: 1%; Figure S5 of the Supplementary File 1). The few samples with a z-score absolute value above 3 for one of the metabolite standards were manually validated: no consistent outlier pattern across all spiked standards nor any outlier location on the PCA score plot were observed for any of those samples.

We further used a set of five metrics to assess the global quality of all six metabolomics datasets: the first two metrics assess the absence of any analytical drift between the samples by using univariate and multivariate approaches. The remaining three scores focus on the quality of the pooled QC samples: we first define a scoring of the QC spread on the PCA score plot, and then include two metrics corresponding to the coefficient of variation (CV)^47,48^ and the intraclass correlation coefficient (ICC)^63^. Each metric, ranging from 0 to 100 is computed as follows (a score of 100 corresponds to the highest quality):

1. Drift Spearman (proportion of features which are not significantly correlated with the injection order): $\left(1-\frac{{p}_{correlated}}{{p}_{total}}\right)\times 100$, where *p*_*correlated*_ is the number of features which are correlated to the injection order (False Discovery Rate: 5%) and *p*_*total*_ is the total number of features
2. Drift PCA (proportion of variance of the injection order which is not explained by the 3 first PCA components): (1−*cos*^2^*α*) × 100, where *a* is the angle between the direction of the injection order and its projection on the PCA hyperspace of the 3 first components; a value of 100 (i.e. *cosα* = 0) corresponds to the absence of correlation with the injection order (the direction of the injection order gradient is orthogonal to the PCA 3D hyperspace)
3. QC spread (spread of the pooled QC samples in the PCA 3D hyperspace): $\left(1-\frac{max{\rm{(}}{d}_{QC}{\rm{)}}}{max{\rm{(}}{d}_{sample}{\rm{)}}}\right)\times 100$, where *max*(*d*_*QC*_) is the Mahalanobis distance to the center of the farthest pooled QC sample in the PCA 3D hyperspace and *max*(*d*_*sample*_) is the distance of the farthest biological sample
4. QC CV (coefficient of variation of the pooled QC intensities): percentage of features with a coefficient of variation of the intensities in the QCs inferior to 30%^47,48^: $\frac{{p}_{CV\le 0.3}}{{p}_{total}}\times 100$, where *p*_*CV* ≤ 0.3_ is the number of features with an intensity CV of less than 30% in the pooled QC; recently, Zhang *et al*. (2020) proposed to further display the cumulative percentage of compounds as a function of CV for a more detailed overview of the dataset quality^63^. The corresponding graphic is therefore provided as the Figure S7 of the Supplementary File 1
5. QC ICC (intraclass correlation coefficient, ICC, between the pooled QC intensities at the most probable abundance^63^): *ICC*_*most probable abundance*_ × 100, the use of the ICC between the pooled QC has been recently proposed as a metric to assess the reliability of the measurements^63^. The cumulative ICCs as a function of the intensities are shown in the Figure S8 of the Supplementary File 1, and the ICC value at the intensity bin containing most compounds (most probable abundance) is used as the quality metric^63^. The *icc* function from the *irr* package^64^ was used with the “twoway” random effect model, “agreement”, and “single” parameters.

**Fig. 7:**
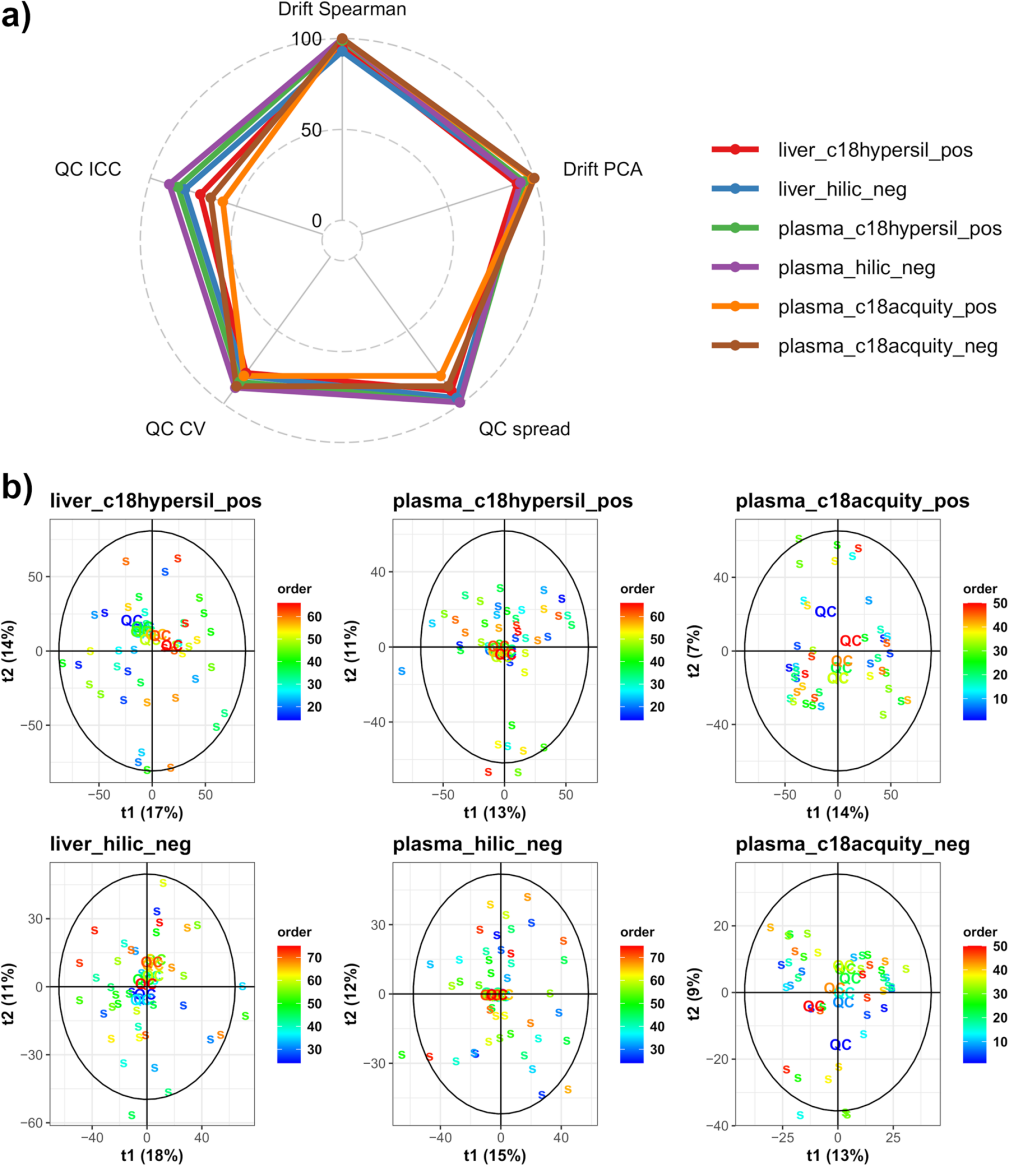
Quality control of the metabolomics datasets. (a) Scores of the datasets according to five metrics visualized as a radar plot. The two “Drift” metrics assess the absence of unwanted variation due to a signal drift by univariate and multivariate approaches, whereas the three “QC” metrics evaluate the quality of the pooled QC samples. CV: coefficient of variation; ICC: intraclass correlation coefficient. (b) PCA score plots displaying the samples and pooled QCs with a color gradient according to the injection order. QC: pooled quality control sample; s: biological sample.

High scores were obtained for all datasets, with an average value of the metrics ranging between 84 and 94% (for the *plasma_c18acquity_pos* and *plasma_hilic_neg* datasets, respectively; Table 1 and Fig. 7). The normalization based on pooled QCs (respectively, on samples) was necessary to achieve a correction of the signal drift for the *plasma_c18hypersil/hilic* datasets (respectively, the *plasma_c18acquity datasets*, Figure S6 of the Supplementary File 1), as assessed by the Drift Spearman and Drift PCA metrics scores above 90%. Furthermore, the three QC metrics of the datasets were high (QC spread, QC CV, QC ICC), even in the absence of signal drift correction or after a normalization based on the samples, with an average value of 83% (min-max: 58–99%). The curves showing the cumulative percentage of compounds as a function of the CVs in the pooled QC samples were similar to those obtained by Zhang *et al*.^63^ with a repeated injection of a single serum sample (Figure S7 of the Supplementary File 1): in all metabolomics datasets, a minimum of 79% of features had a CV below 30%. The cumulative ICC as a function of the intensity in the pooled QCs was similar to those from Zhang *et al*.^63^, with an ICC value at the most probable intensity between 71% and 89%, except for the *plasma_c18acquity* datasets which had slightly lower values (58% and 65%), confirming that although the pooled QCs were less reliable in these latter datasets, the overall quality of all metabolomics datasets was high (Figure S8 of the Supplementary File 1). Finally, the distribution of CVs in the biological samples were similar for all genotypes (Figure S9 of the Supplementary File 1), as observed in proteomics (Fig. 6 right).

**Table 1:** Quality metrics for the metabolomics datasets.

	Drift Spearman	Drift PCA	QC spread	QC CV	QC ICC
liver_c18hypersil_pos	95	90	91	79	71
liver_hilic_neg	93	93	96	81	80
plasma_c18hypersil_pos	99	94	99	85	84
plasma_hilic_neg	100	92	99	89	89
plasma_c18acquity_pos	100	99	81	81	58
plasma_c18acquity_neg	100	100	88	88	65

PCA: principal component analysis; QC: pooled quality control sample; CV: coefficient of variation; ICC: intraclass correlation coefficient.

### Sex-specific validation

Individual features with significant difference in means between males and females were detected in all *ProMetIS* datasets, as it is often observed in phenotyping studies (from 14% to 40% of the total number of features, depending on the dataset; limma test, 5% threshold, Benjamini and Hochberg correction of the False Discovery Rate). We therefore checked the absence of any systematic bias related to sex on feature intensities, CVs and imputation (Figures S10, S11 and S12, respectively, of the Supplementary File 1).

## Usage Notes

The *tutorial* vignette from the *ProMetIS* package^27^ describes in detail how to load the data and provides examples of single omics and integrative data analysis of the datasets with R packages, such as univariate hypothesis testing (*phenomis*; https://github.com/SciDoPhenIA/phenomis), orthogonal partial least-square – discriminant analysis (*ropls*)^48^, feature selection (*biosigner*)^65^, sparse multi-block PLS-DA modeling (*mixOmics*)^66^.

Alternatively, the proteomics and metabolomics data may also be readily loaded and analyzed online by using the Workflow4Metabolomics^43^ and ProteoRE^67^ platforms. These resources are based on the Galaxy environment to facilitate workflow management^68^, and include many modules for statistical analysis and annotation^60,69^.

## Supplementary information

Supplementary File 1

Supplementary File 2

Supplementary File 3

## Data Availability

The dataset and the source code for the data analysis are publicly available as the *ProMetIS* R package^27^ on GitHub (https://github.com/IFB-ElixirFr/ProMetIS). The package includes several vignettes describing the pre-processing of the data as well as the tables and figures from this article (*6_article_data*).
