# Supplementary material for: ProMetIS, deep phenotyping of mouse models by combined proteomics and metabolomics analysis
Source: Sci Data. 2021 Dec 3;8:311. doi: 10.1038/s41597-021-01095-3 (PMC8642540; doi:10.1038/s41597-021-01095-3)
Supplement: Supplementary file 1 [file 41597_2021_1095_MOESM1_ESM.docx]

Supplementary Information

ProMetIS, deep phenotyping of mouse models by combined proteomics and metabolomics analysis

Alyssa Imbert, Magali Rompais, Mohammed Selloum, Florence Castelli, Emmanuelle Mouton-Barbosa, Marion Brandolini-Bunlon, Emeline Chu-Van, Charlotte Joly, Aurélie Hirschler, Pierrick Roger, Thomas Burger, Sophie Leblanc, Tania Sorg, Sadia Ouzia, Yves Vandenbrouck, Claudine Médigue, Christophe Junot, Myriam Ferro, Estelle Pujos-Guillot, Anne Gonzalez de Peredo, François Fenaille, Christine Carapito, Yann Herault, and Etienne A. Thévenot

The supplementary information is organized as follows:

- *Supplementary File 1 (this file)*: *Word* document with the additional figures supporting the *Technical Validation* section of the proteomics and metabolomics data; by default, only the processing step (respectively, the processing and the signal drift correction steps) have been applied to the proteomics (respectively, the metabolomics) data sets at that stage, unless additional steps of the post-processing workflow are explicitly stated in the figure legend (e.g., log2 transformation in the Figures S1, S5 and S10; post-processing of the proteomics data in the Figure S12)
- *Supplementary File 2*: *Excel* file containing 9 tabs for the tables of intensities of each dataset, after the post-processing step (in particular, intensities are log2 transformed, except for the preclinical dataset), preceded by 1 tab for the sample metadata (e.g., correspondence between the abbreviations of the sample names used in the datasets and the IMPC reference IDs, genotypes and phenotypes)
- *Supplementary File 3*: *Excel* file with the list of the procedures used for the preclinical phenotyping at PHENOMIN ICS, and corresponding links to the IMPReSS database

[Proteomics 3](#_Toc82717756)

[Fig. S1: Quality control of the LC-MS analytical sequences using a spiked-in internal standard, i.e., 11 iRT peptides (iRT kit; Biognosys) spiked in all the analyzed liver (top) and plasma (bottom) samples. Log2 of peptide raw intensities are plotted in the chronological order of data acquisition. Median CV of intensity values across all liver (respectively, plasma) samples injections is 40% (respectively, 31%). 3](#_Toc82717757)

[Fig. S2: Quality control of the liver LC-MS variability in the pool standards (left) and biological samples (right), using three metrics: numbers of MS/MS scans, identified peptides and proteins. 3](#_Toc82717758)

[Fig. S3: Quality control of the plasma LC-MS variability in the pool standards (left) and biological samples (right) using three metrics: numbers of MS/MS scans, identified peptides and proteins. 4](#_Toc82717759)

[Fig. S4: Quality control of the LC-MS variability in liver (right) and plasma (left) pools. Dot-plots of the protein raw intensities are shown for each pair of pools with associated correlation factor R2 values. 4](#_Toc82717760)

[Metabolomics 5](#_Toc82717761)

[Fig. S5: Quality control of the intensities of the metabolite standards (Hypersil C18 and HILIC datasets). Internal standards (IS) are spiked in the samples before the extraction step whereas external standards (ExS) are added just before the LC-HRMS analysis (Boudah *et al.*, 2014). Shaded bands correspond to the average $\pm$ 2$\times$standard deviation. AMPA: 2-amino-3-(3-hydroxy-5-methyl-isoxazol-4-yl)propanoic acid; MCPA: 2-methyl-4-chlorophenoxyacetic acid. 6](#_Toc82717762)

[Fig. S6: Quality metrics of the metabolomics datasets without signal drift correction (“none”), or with a *loess* normalization based on pooled QCs (“pool”) or samples (“sample”). CV: coefficient of variation; ICC: intraclass correlation coefficient. 7](#_Toc82717763)

[Fig. S7: Cumulative percentage of compounds as a function of the Coefficient of Variation in the pooled QC samples. 8](#_Toc82717764)

[Fig. S8: Intraclass correlation coefficient (ICC) in pooled QC samples as a function of the cumulative intensity. The (log10) intensity is centered on the bin containing most of the compounds (most probable abundance; Zhang *et al.*, 2020). 8](#_Toc82717765)

[Fig. S9: Coefficient of variations of the pooled QC, wild-type and knock-out mice samples in the metabolomics datasets. 9](#_Toc82717766)

[Sex-specific validation 10](#_Toc82717767)

[Fig. S10: Variable intensities in each sample from each omic dataset, colored according to genotype and sex. 10](#_Toc82717768)

[Fig. S11: CVs of the variable intensities according to genotype and sex. 11](#_Toc82717769)

[Fig. S12: CVs of the post-processed protein intensities with (top) or without (bottom) imputation, according to genotype and sex. 12](#_Toc82717770)

[References 13](#_Toc82717771)

## Proteomics

| Intensity (log2) | liver | 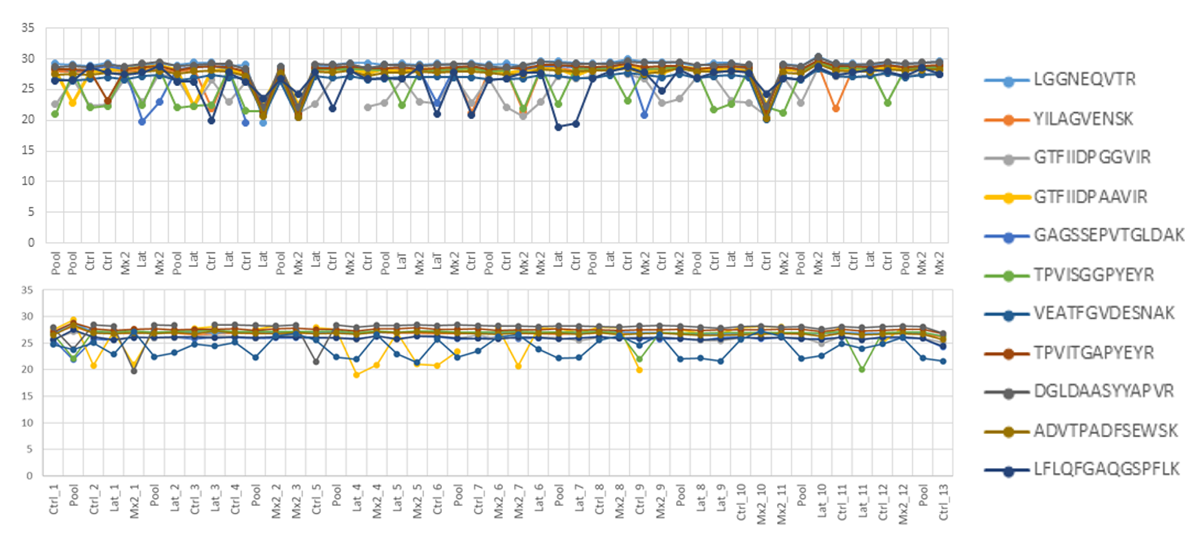 |
| --- | --- | --- |
|  | plasma |  |

### Fig. S1: Quality control of the LC-MS analytical sequences using a spiked-in internal standard, i.e., 11 iRT peptides (iRT kit; Biognosys) spiked in all the analyzed liver (top) and plasma (bottom) samples. Log2 of peptide raw intensities are plotted in the chronological order of data acquisition. Median CV of intensity values across all liver (respectively, plasma) samples injections is 40% (respectively, 31%).


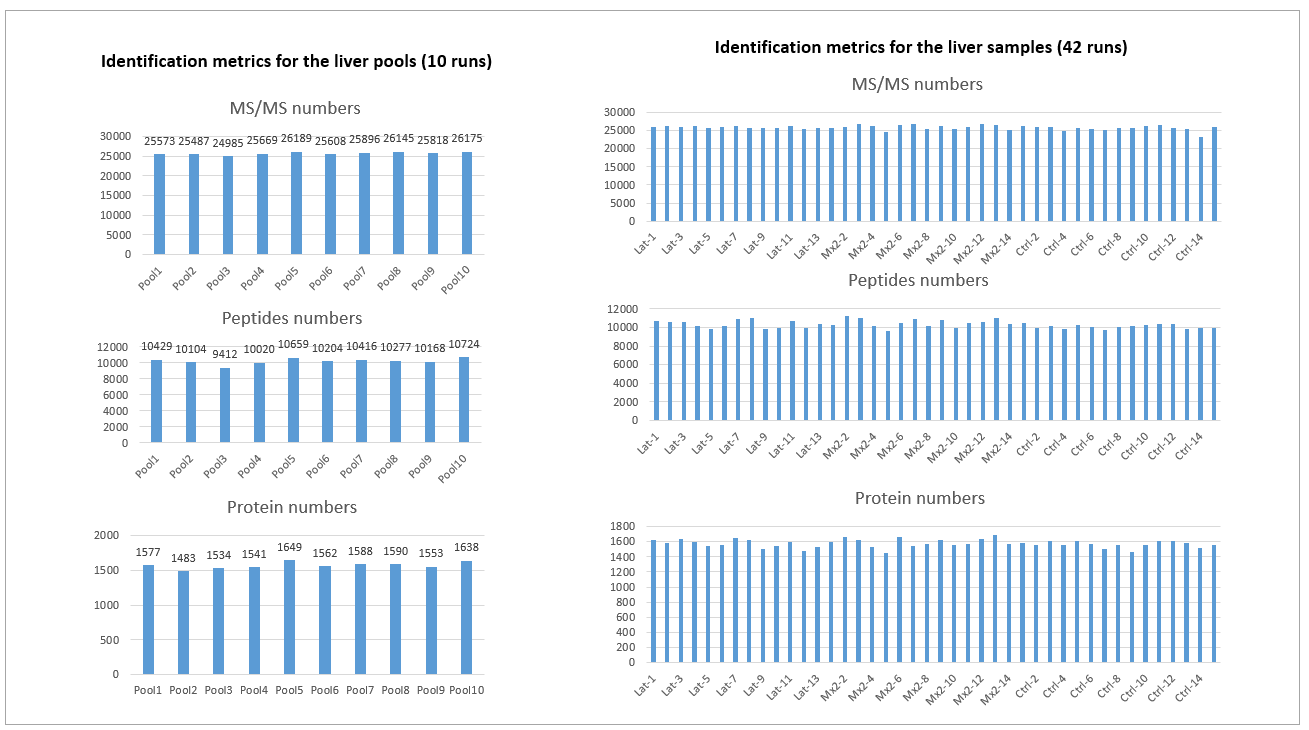


### Fig. S2: Quality control of the liver LC-MS variability in the pool standards (left) and biological samples (right), using three metrics: numbers of MS/MS scans, identified peptides and proteins.


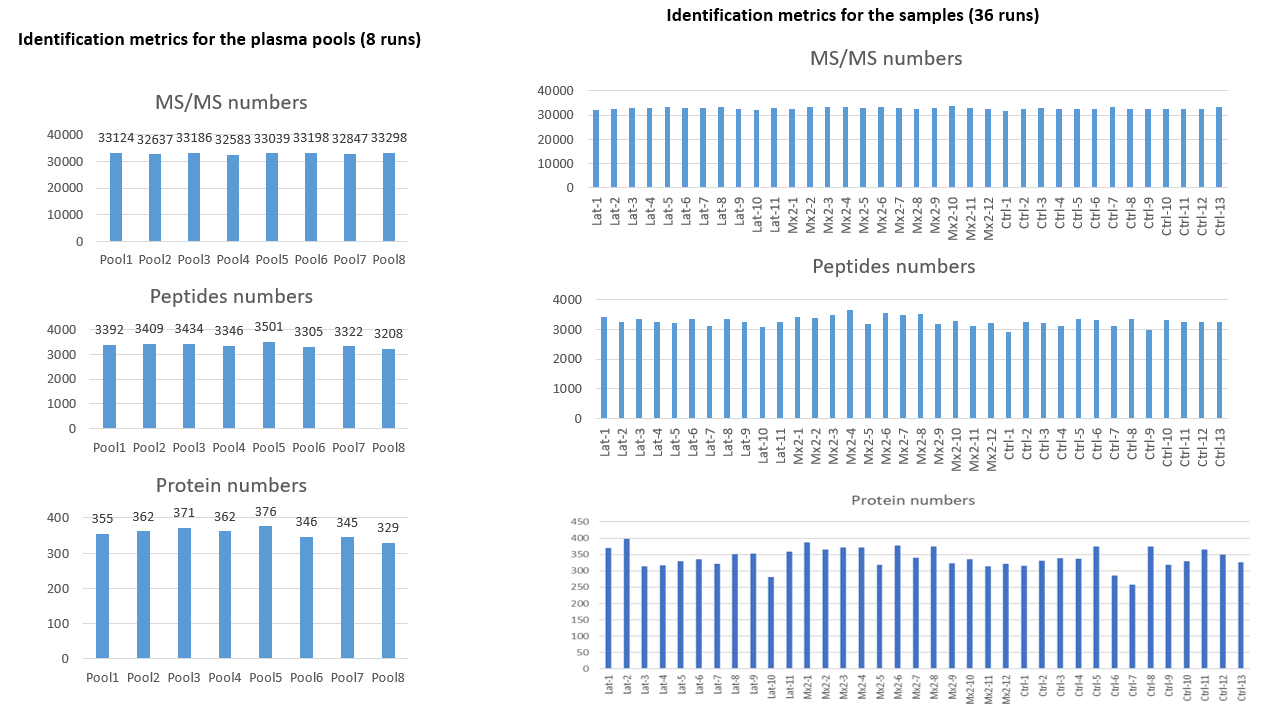


### Fig. S3: Quality control of the plasma LC-MS variability in the pool standards (left) and biological samples (right) using three metrics: numbers of MS/MS scans, identified peptides and proteins.


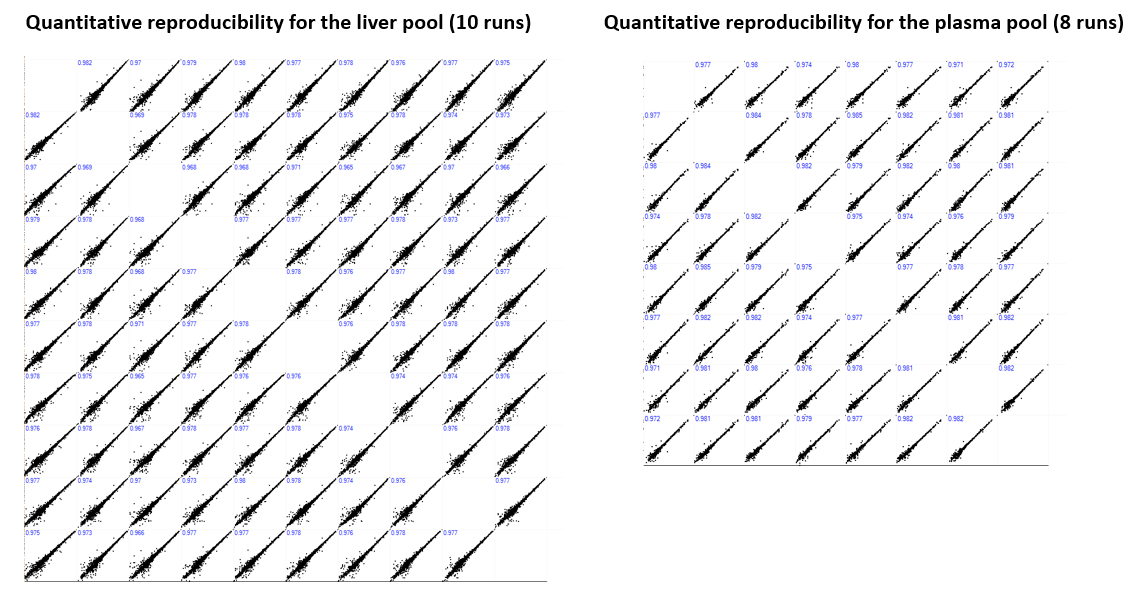


### Fig. S4: Quality control of the LC-MS variability in liver (right) and plasma (left) pools. Dot-plots of the protein raw intensities are shown for each pair of pools with associated correlation factor R2 values.

## Metabolomics


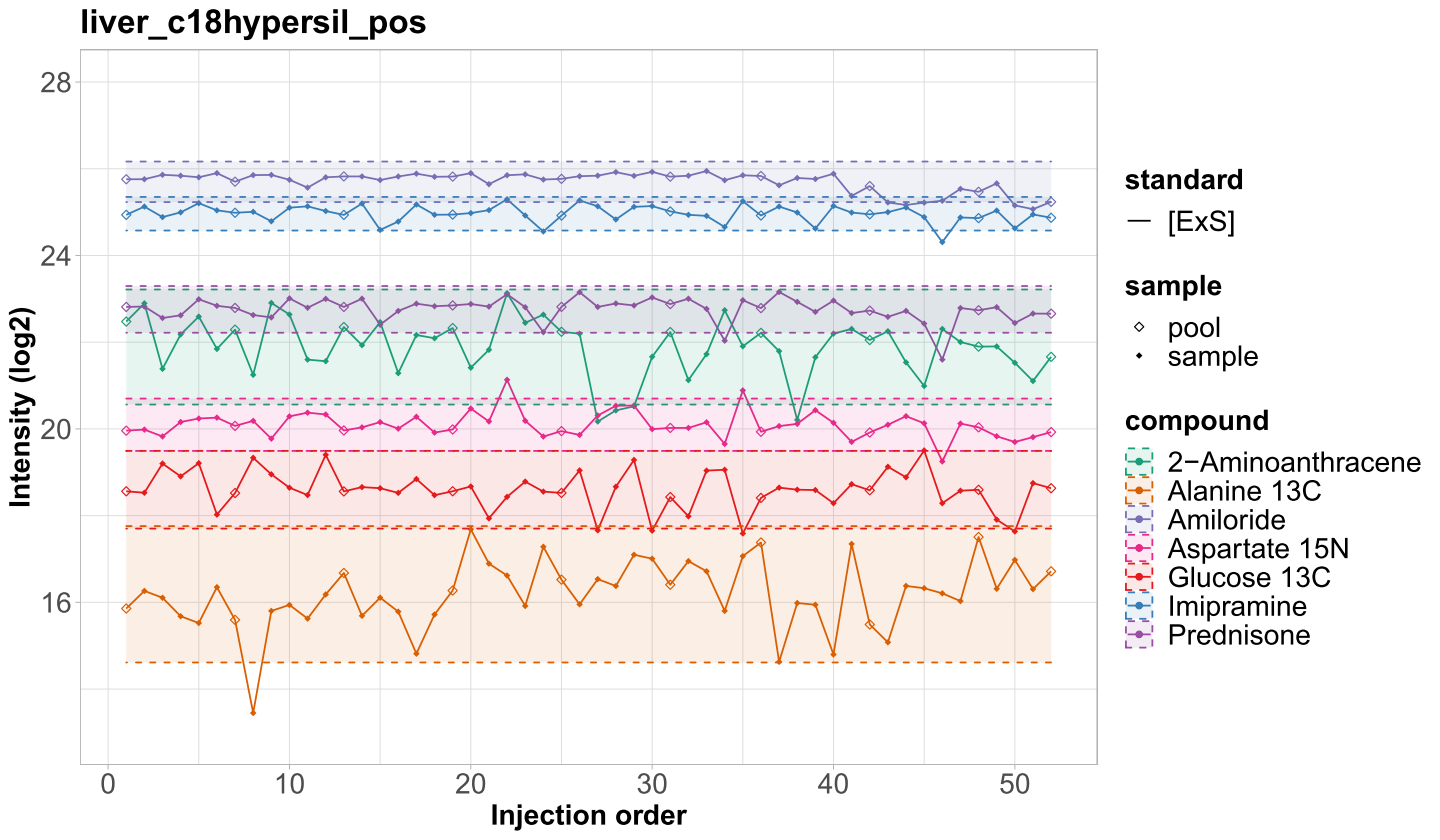


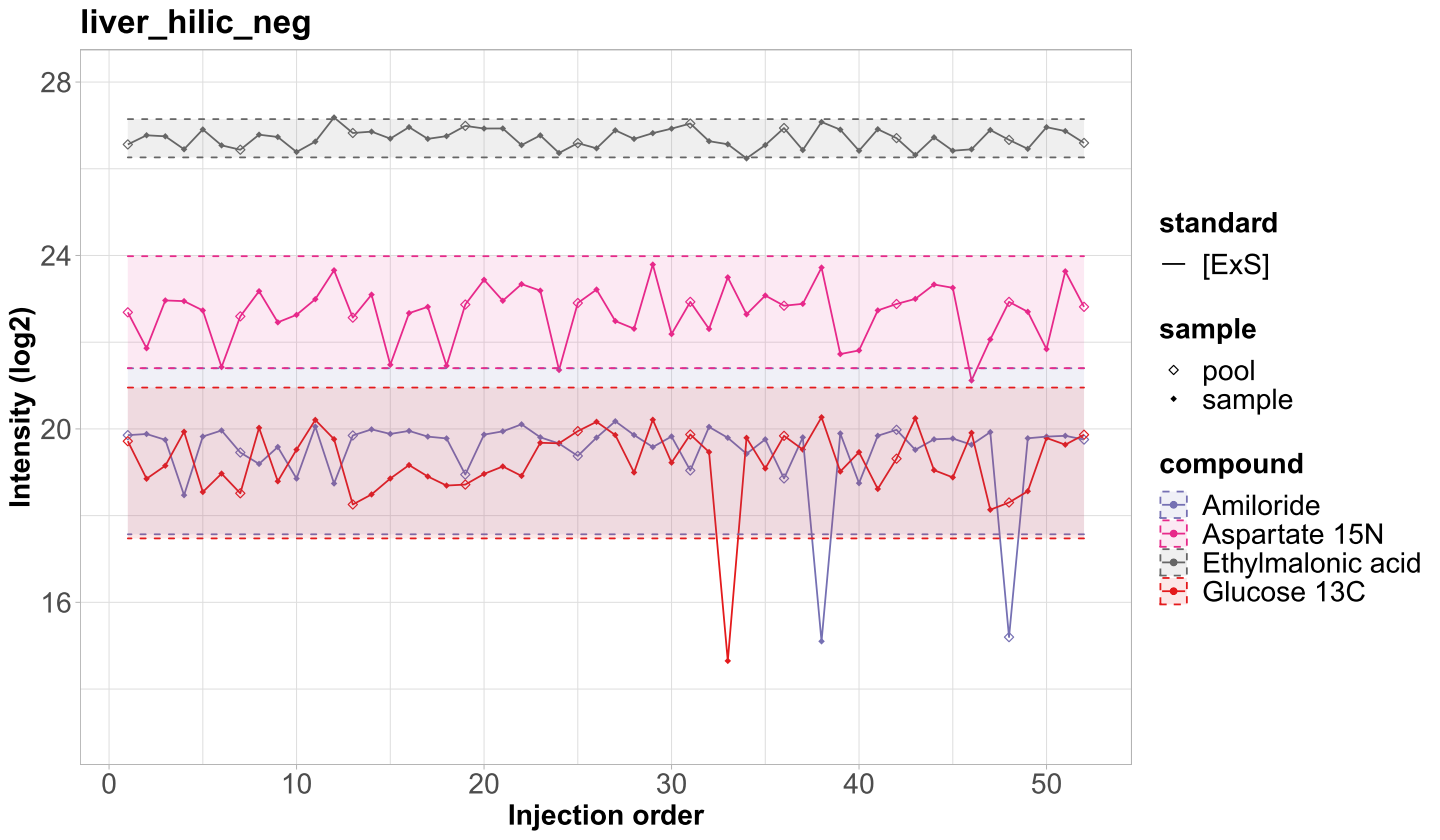


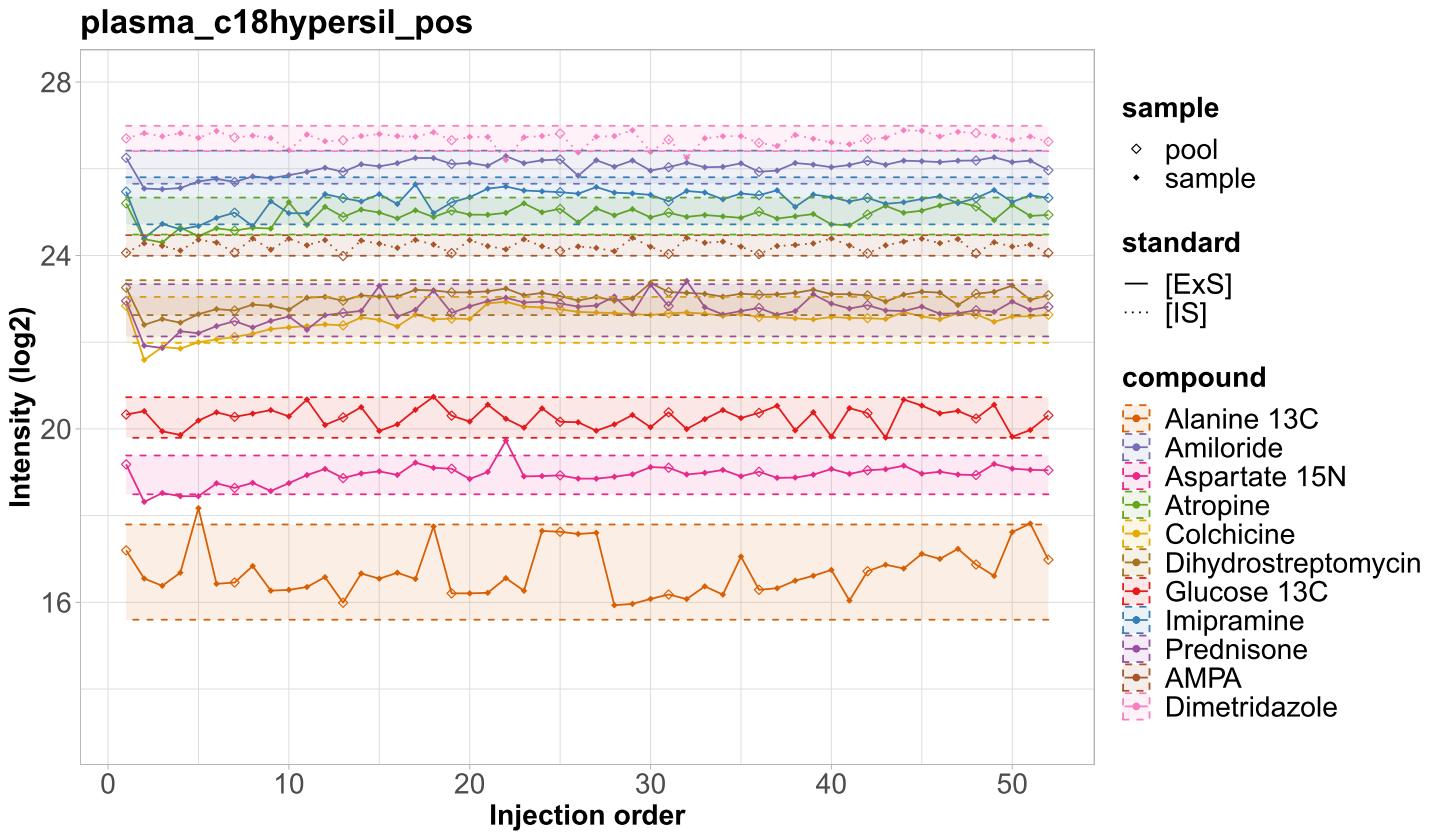


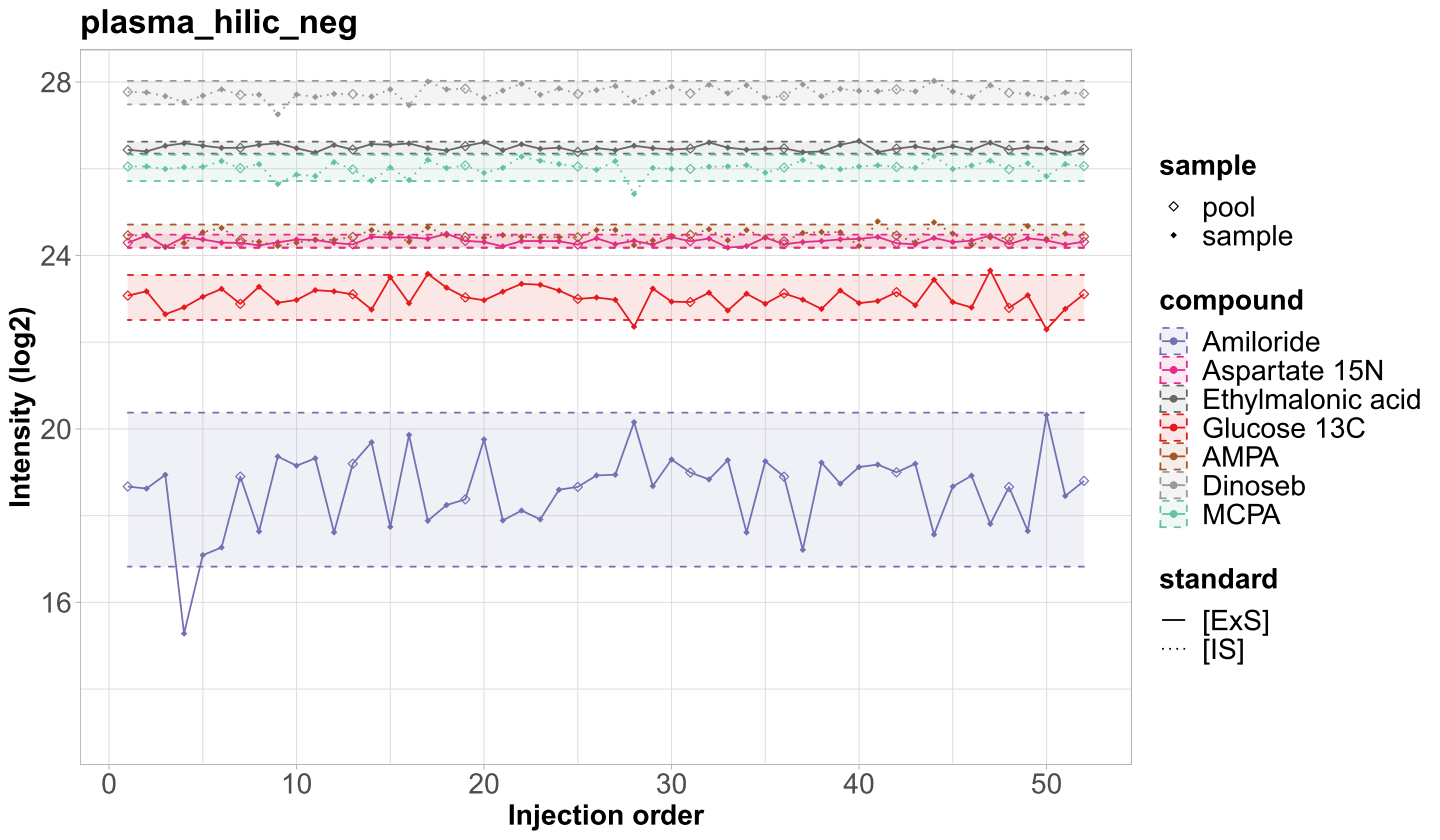


### Fig. S5: Quality control of the intensities of the metabolite standards (Hypersil C18 and HILIC datasets). Internal standards (IS) are spiked in the samples before the extraction step whereas external standards (ExS) are added just before the LC-HRMS analysis (Boudah *et al.*, 2014). Shaded bands correspond to the average $\pm$ 2$\times$standard deviation. AMPA: 2-amino-3-(3-hydroxy-5-methyl-isoxazol-4-yl)propanoic acid; MCPA: 2-methyl-4-chlorophenoxyacetic acid.


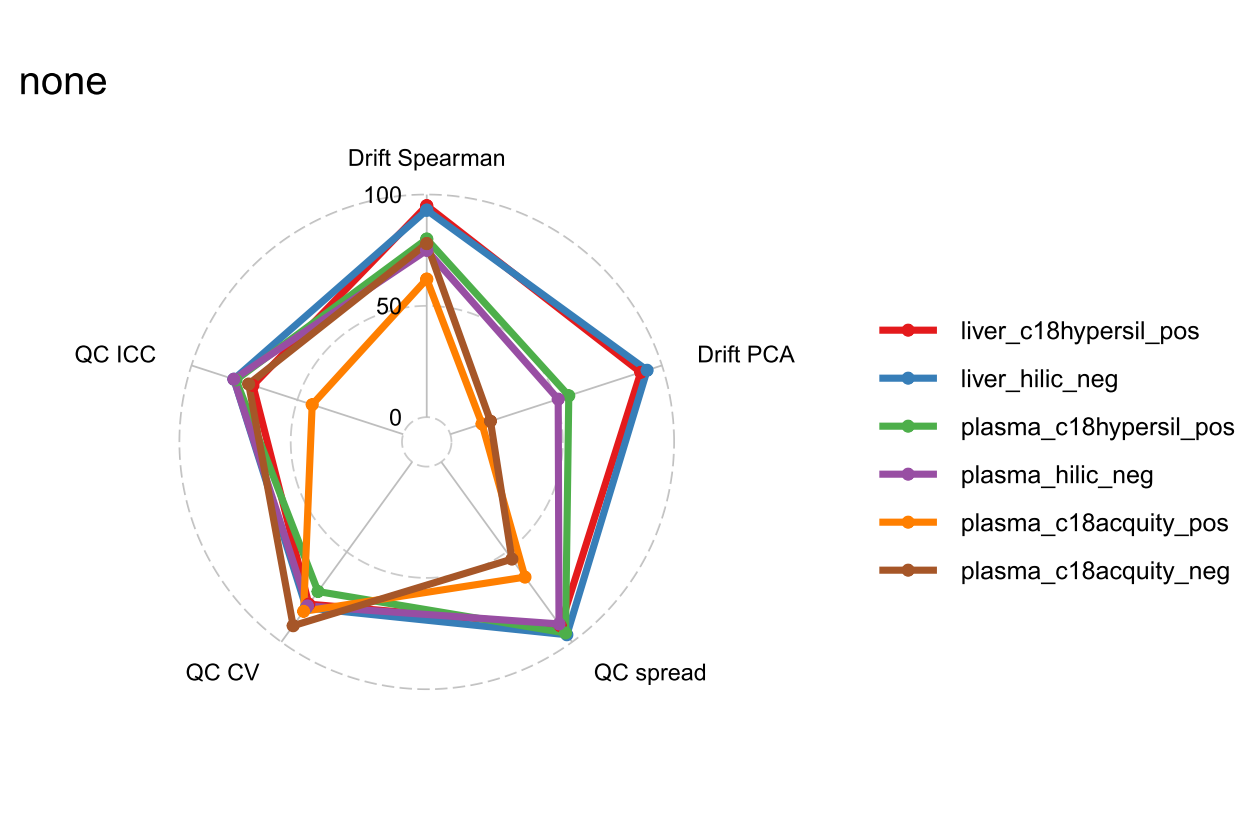

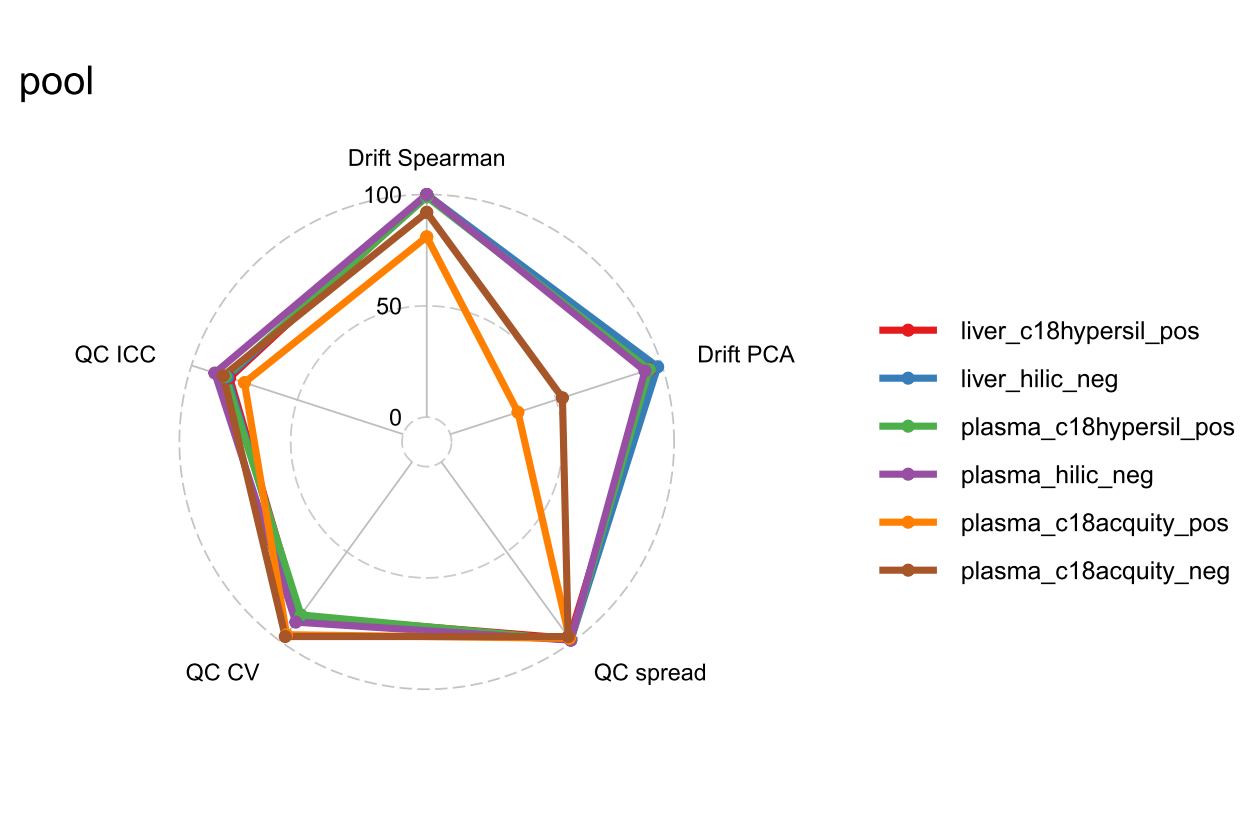


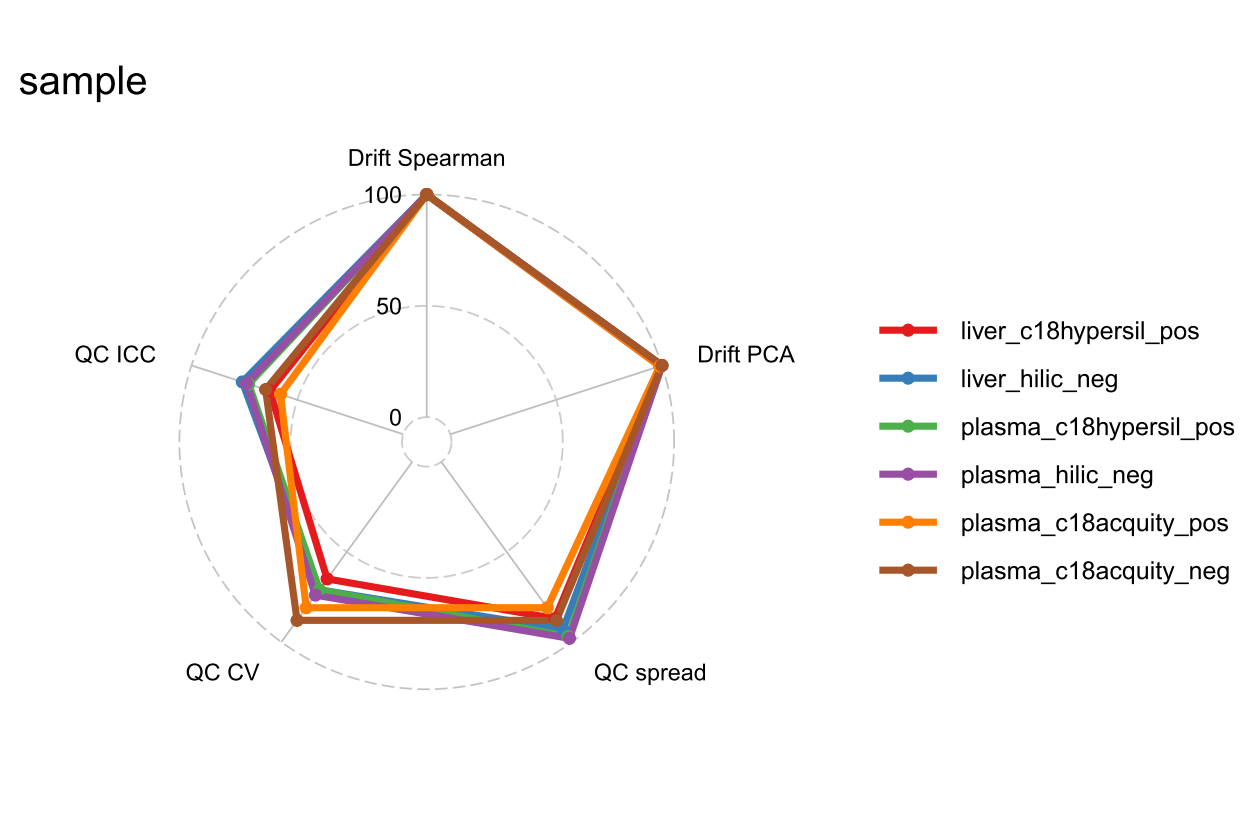


### Fig. S6: Quality metrics of the metabolomics datasets without signal drift correction (“none”), or with a *loess* normalization based on pooled QCs (“pool”) or samples (“sample”). CV: coefficient of variation; ICC: intraclass correlation coefficient.


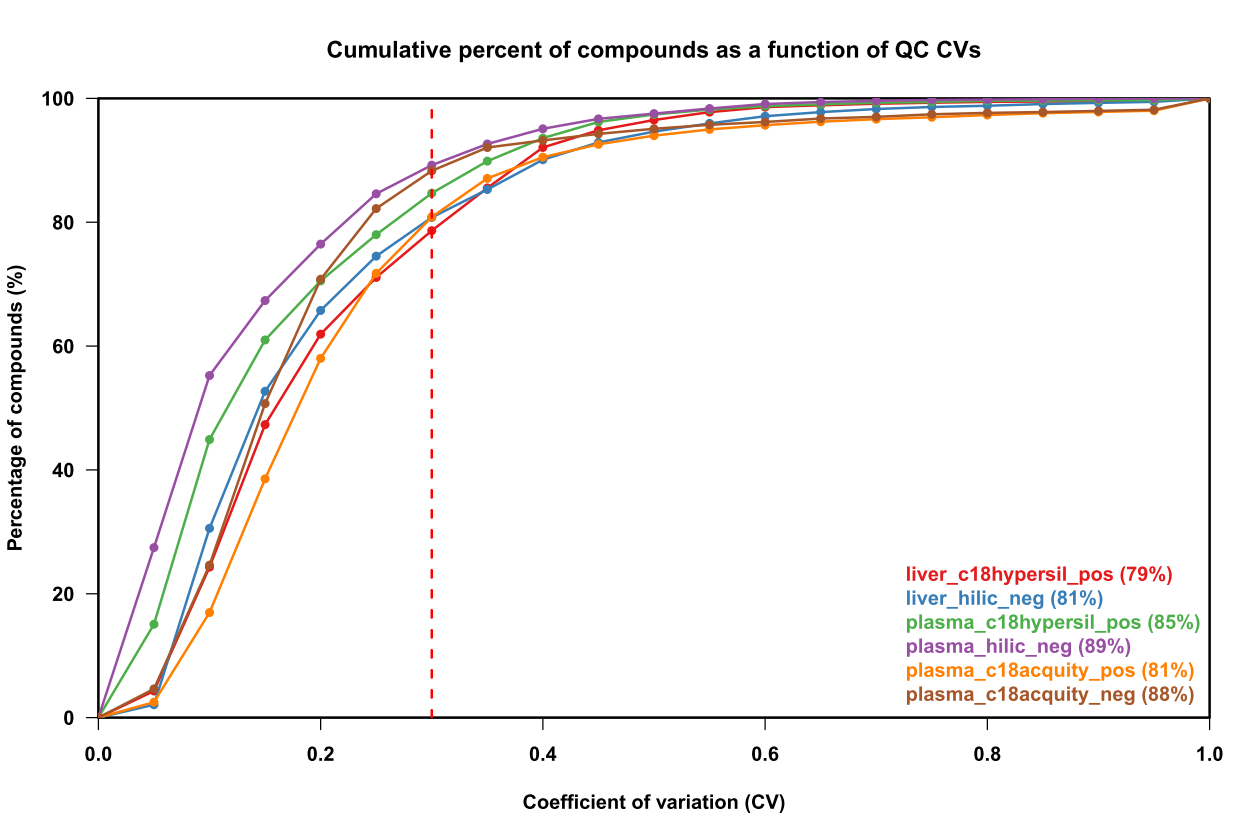


### Fig. S7: Cumulative percentage of compounds as a function of the Coefficient of Variation in the pooled QC samples.


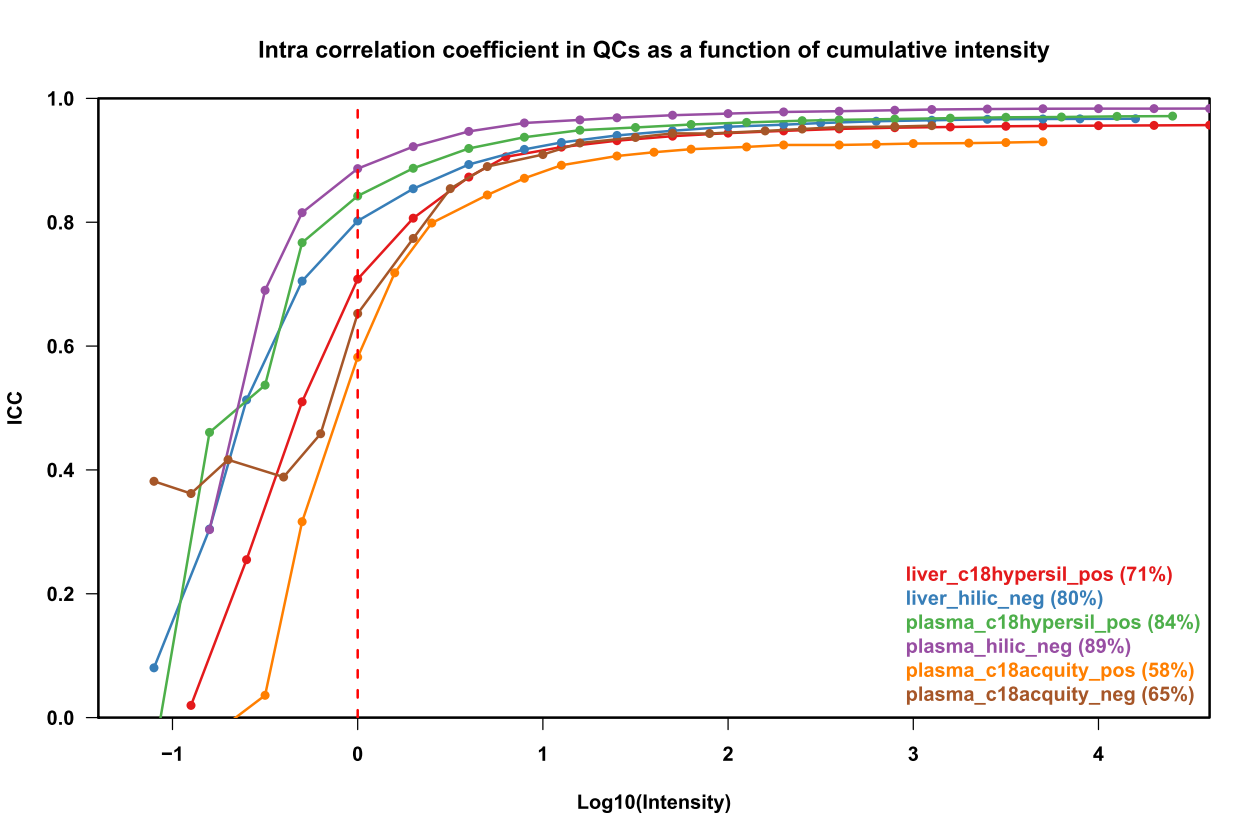


### Fig. S8: Intraclass correlation coefficient (ICC) in pooled QC samples as a function of the cumulative intensity. The (log10) intensity is centered on the bin containing most of the compounds (most probable abundance; Zhang *et al.*, 2020).


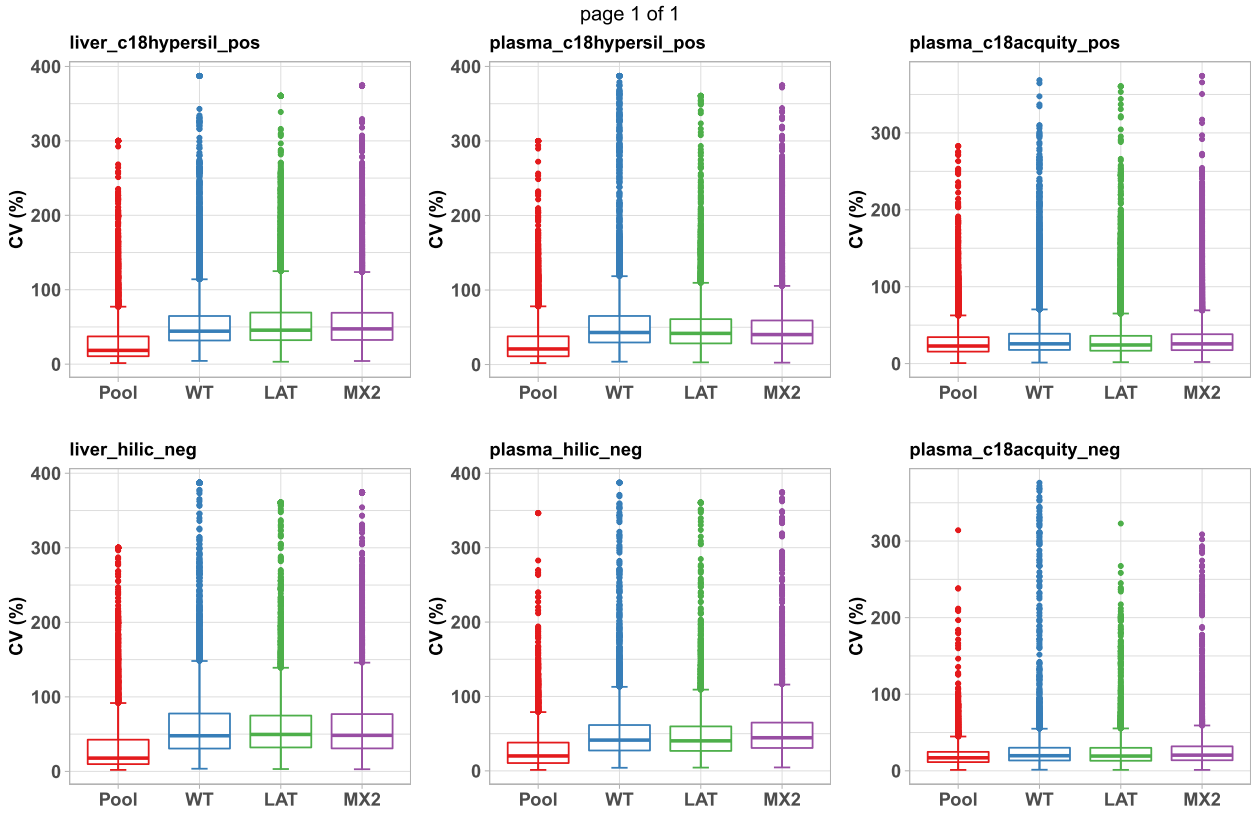


### Fig. S9: Coefficient of variations of the pooled QC, wild-type and knock-out mice samples in the metabolomics datasets.

## Sex-specific validation


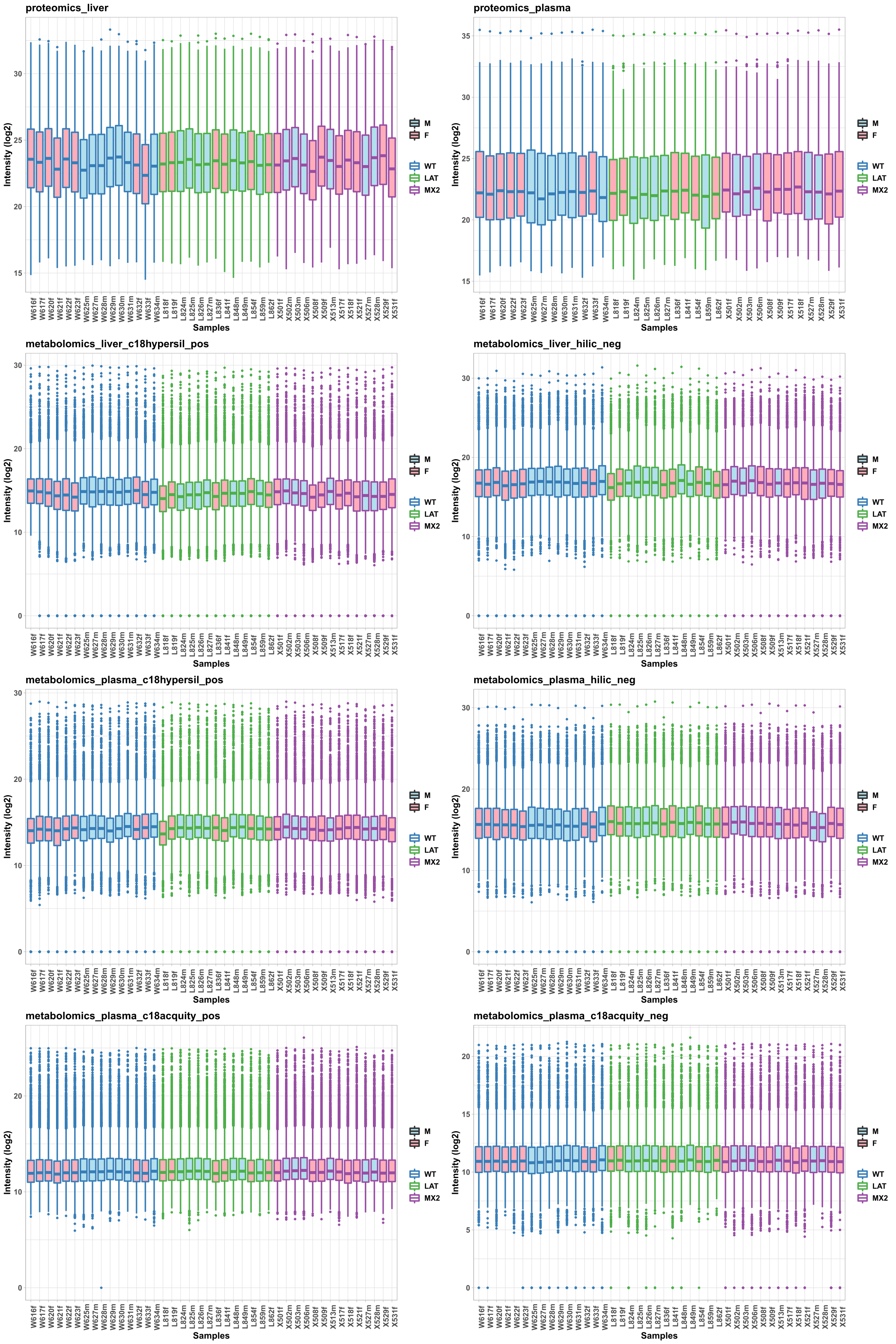


### Fig. S10: Variable intensities in each sample from each omic dataset, colored according to genotype and sex.


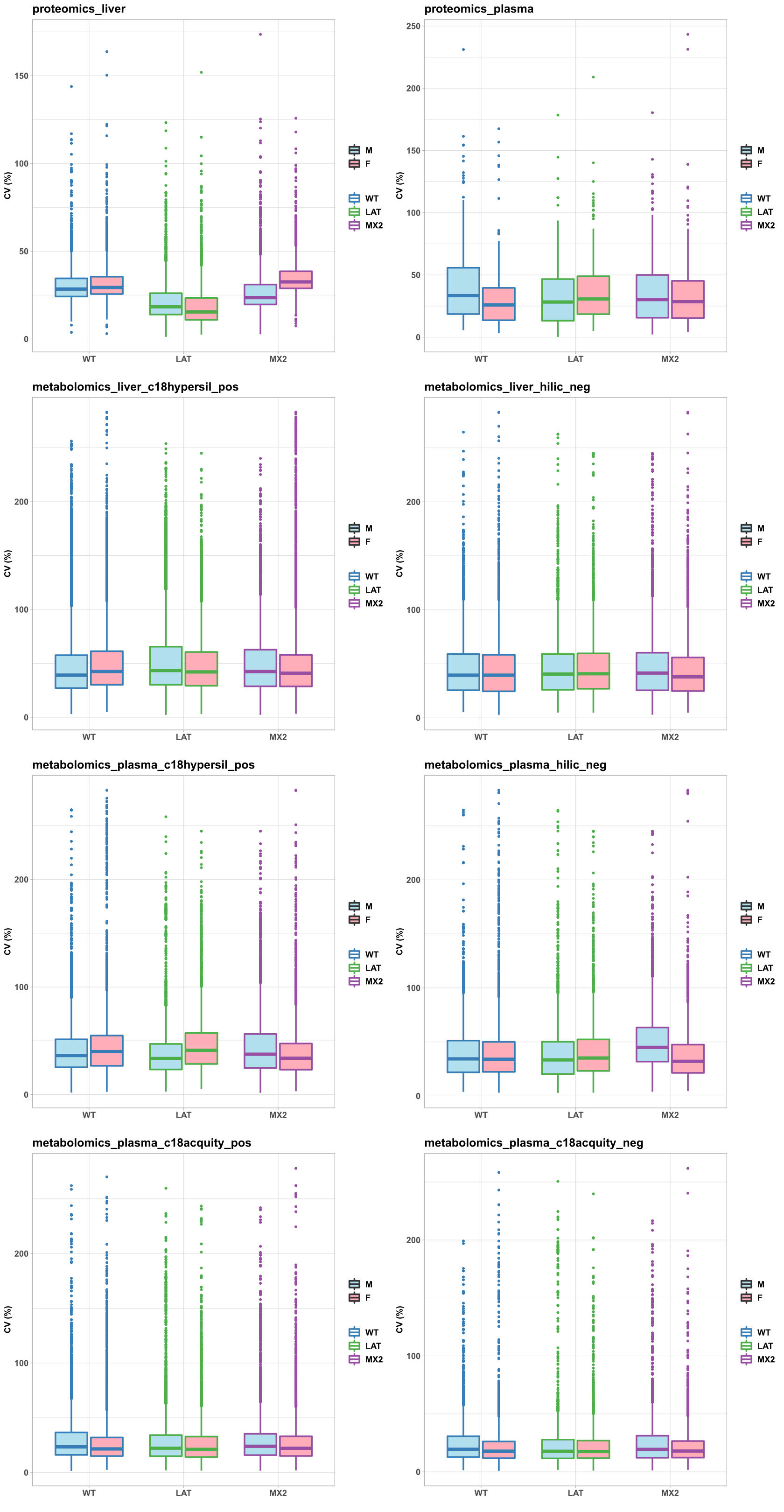


### Fig. S11: CVs of the variable intensities according to genotype and sex.


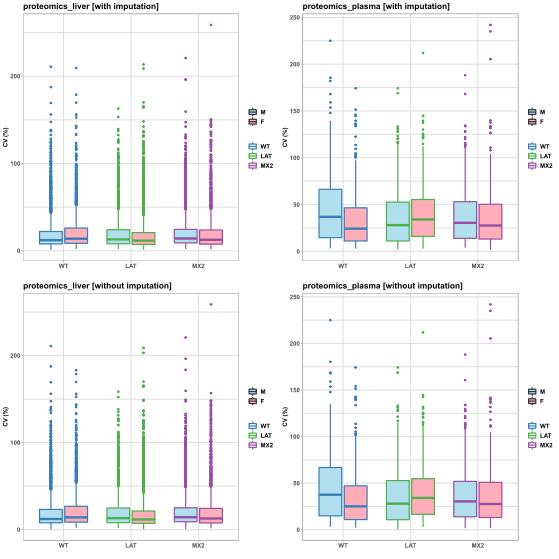


### Fig. S12: CVs of the post-processed protein intensities with (top) or without (bottom) imputation, according to genotype and sex.

## References

Boudah, S. *et al.* Annotation of the human serum metabolome by coupling three liquid chromatography methods to high-resolution mass spectrometry. *J Chrom B* **966**, 34–47, <https://doi.org/https://doi.org/10.1016/j.jchromb.2014.04.025> (2014)

Zhang, X., Dong, J., & Raftery, D. (2020). Five easy metrics of data quality for LC-MS based global metabolomics. *Anal Chem* **92**, 12925–12933. <https://doi.org/10.1021/acs.analchem.0c01493> (2020).
